# Phylogeny and comparative chloroplast genomics reveal a rare new short-styled homostylous species of *Primula* (Primulaceae) from southwestern China

**DOI:** 10.3389/fpls.2026.1934280

**Published:** 2026-09-29

**Authors:** Qing-qing Ye, Yong-lin Liu, Ying Guo, Qiang Deng, Wei Zhou, Zhi-kun Wu

**Affiliations:** 1 Department of Pharmacy, Guizhou University of Traditional Chinese Medicine, Guiyang, China; 2 Panzhou Bureau of Natural Resources, Panzhou, China; 3 Panzhou Convergence Media Center, Panzhou, China; 4 Germplasm Bank of Wild Species & Yunnan Key Laboratory of Crop Wild Relatives Omics, Kunming Institute of Botany, Chinese Academy of Sciences, Kunming, China

**Keywords:** chloroplast genomics, molecular phylogeny, morphological taxonomy, *Primula panzhouensis*, *Primula sect*. *Petiolares*, short-styled homostylous species

## Abstract

Based on comparative chloroplast genomes (cp genomes) and phylogenetic analyses integrated with morphological evidence, this study investigated the taxonomic status and phylogenetic relationships of an extremely rare short-styled homostylous lineage of *Primula* from southwestern China, providing new molecular insights into the delimitation of this complex group. Complete cp genomes were sequenced and comparatively analyzed for one putative new taxon, *Primula panzhouensis*, and 15 representative samples within *Sect. Petiolares* from southwestern China. The cp genome characteristics, simple sequence repeat (SSR) patterns, codon usage bias, inverted repeat (IR) boundary variations, and nucleotide diversity patterns were systematically evaluated. Maximum likelihood (ML) and Bayesian inference (BI) analyses were conducted based on complete cp genome sequences, and an integrative taxonomic assessment was performed by combining cp genomic evidence with morphological comparisons. All 16 cp genomes exhibited a typical quadripartite structure, with genome sizes ranging from 151,076 to 153,420 bp. The overall cp genome structure and gene composition were highly conserved, although interspecific variations were detected in IR boundary regions, SSR distributions, and highly variable regions, including *trnH-psbA* and *ycf1*. The sequenced cp genome of *P. panzhouensis* exhibited several distinctive structural features and sequence variation patterns compared with closely related taxa. ML and BI analyses consistently recovered *P. panzhouensis* as a distinct cp lineage within *Sect. Petiolares* with strong support. Morphological comparisons further demonstrated that *P. panzhouensis* possesses the short-styled homostyly trait and can be distinguished from related taxa by differences in floral style structure, corolla morphology, scape length, and leaf characteristics. Integrating cp genomic evidence, phylogenetic relationships, and morphological traits, our results support the recognition of *P. panzhouensis* as a distinct taxonomic entity, which is herein described as a new species, *Primula panzhouensis* Z.K.Wu, sp. nov. This study demonstrates that comparative cp genomics integrated with phylogenetic analyses can effectively assist in resolving lineage relationships and taxonomic boundaries in complex groups of *Primula*. It also identifies an extremely rare short-styled homostylous species, providing valuable material for further investigating the evolution of floral diversity and its potential evolutionary significance in *Primula*.

## 1 Introduction

Understanding speciation processes and phylogenetic relationships is fundamental to elucidating the origin and maintenance of biodiversity. In angiosperms, the chloroplast genome (cp genome), characterized by predominantly maternal inheritance, a relatively conserved structure, and a moderate rate of molecular evolution, has been widely used in species identification, cryptic species detection, and phylogenetic reconstruction (Cauz-Santos, 2025). With the rapid advancement of high-throughput sequencing technologies, comparative cp genomics has markedly improved phylogenetic resolution, facilitating the identification of cryptic taxa that are difficult to distinguish based solely on morphology (Tian et al., 2023; Huang et al., 2024). Particularly in rapidly radiating lineages, cp genome-based analyses, including assessments of structural variations (e.g., expansions and contractions of inverted repeat regions), simple sequence repeat (SSR) distributions, and highly variable regions, have become important sources of molecular evidence for investigating lineage diversification and evaluating species boundaries (Xu et al., 2020; Xie et al., 2023). However, cp genomes represent only a single cytoplasmic genetic component of plant evolutionary history, and cp genome-based phylogenetic signals may be affected by processes such as chloroplast capture, historical gene flow, and incomplete lineage sorting. Therefore, cp genome evidence should be integrated with morphological traits and other independent lines of evidence for a comprehensive evaluation of species boundaries and taxonomic status (Wang et al., 2026).

The genus *Primula* (Primulaceae) represents one of the most species-rich and geographically structured lineages of angiosperms, with a broad distribution across temperate and alpine regions of the Northern Hemisphere. Southwestern China is recognized as a major center of diversification for *Primula*, harboring exceptionally high species richness and endemism (Hu, 1990; Richards, 2002). Species within *Primula* exhibit remarkable morphological diversity and are characterized by heterostyly, a specialized reproductive system that facilitates outcrossing through the reciprocal positioning of stigmas and anthers, thereby promoting pollination efficiency and contributing to the maintenance of genetic diversity (Richards, 2002; Keller et al., 2014). However, delimiting species boundaries among closely related taxa remains challenging, particularly in rapidly diversifying lineages such as Sect. *Obconicolisteri* and Sect. *Petiolares* (Li, 2022; Wu et al., 2023), where cryptic diversity is frequently encountered and traditional morphological approaches alone may be insufficient for accurate taxonomic resolution.

Recent molecular genetic studies have elucidated the genetic basis underlying heterostyly in *Primula*, revealing that the *S*-locus supergene system plays a central role in maintaining floral polymorphism. Several key regulatory genes within the *S*-locus, including *CYP734A50*, have been demonstrated to contribute to the maintenance of distyly by regulating reciprocal positioning of floral organs (Huu et al., 2016; Li et al., 2016). Notably, degeneration, loss, or structural rearrangement of the *S*-locus can result in transitions from heterostyly to homostyly, leading to repeated evolutionary shifts in reproductive strategies across different lineages. Compared with the widespread occurrence of heterostyly, which is present in more than 90% of *Primula* species (Richards, 2002), homostylous taxa are relatively uncommon. Although both short-styled homostyly and long-styled homostyly have been documented in *Primula*, long-styled homostyly is more frequently observed (Charlesworth and Charlesworth, 1979). This difference may be explained by the distinct genetic consequences of *S*-locus disruption. In long-styled homostylous flowers, disruption of *CYP734A50* (*CYPT*) results in both reduced herkogamy and the breakdown of self-incompatibility, thereby facilitating autonomous self-pollination and the production of selfed seeds (Piper et al., 1986; Huu et al., 2016, Huu et al., 2020, Huu et al., 2022). In contrast, short-styled homostyles with disrupted *GLOT* but functional *CYPT* retain self-incompatibility despite reduced herkogamy and therefore cannot produce selfed seeds (Huu et al., 2016). Consequently, the reproductive performance of short-styled homostylous forms under natural conditions and their evolutionary significance in the diversification of *Primula* remain poorly understood and require further investigation through field observations and phylogenetic evidence.

The karst regions of southwestern China have long been recognized as key areas for investigating species diversification and the formation of endemism due to their highly fragmented landscapes and diverse microhabitats. The complex topography and heterogeneous ecological environments in this region are considered to have facilitated localized lineage divergence and the establishment of regional endemism, providing an important evolutionary context for studying plant diversification and the generation of biodiversity (Chen et al., 2019; Gu et al., 2024). Since the publication of the *Flora Reipublicae Popularis Sinicae*, more than 60 new *Primula* taxa have been described from China, most of which originate from these regions. Moreover, *Primula* species distributed in the karst landscapes and alpine canyon systems of southwestern China exhibit pronounced patterns of local endemism. However, the phylogenetic relationships, taxonomic boundaries, and evolutionary histories of narrowly distributed *Primula* lineages in the karst regions of southwestern China remain insufficiently understood. Comparative cp genome analyses combined with phylogenomic approaches provide promising opportunities to elucidate lineage relationships, identify potential cryptic taxonomic entities, and improve our understanding of the evolutionary processes underlying regional plant diversification.

Although previous studies have explored the formation of reproductive diversity in *Primula* from perspectives including *S*-locus evolution, heterostylous system transitions, and ecological adaptation, the phylogenetic position, evolutionary significance, and origin of the short-styled homostylous type remain poorly understood. The putative new species *Primula panzhouensis* provides an important opportunity to investigate this issue. Based on nearly four years of continuous field observations since its discovery, all individuals examined in the natural population exhibited a short-styled homostylous phenotype and were capable of producing seeds under natural conditions, suggesting that this floral type may possess the potential to maintain reproduction in natural populations. This distinctive reproductive phenotype expands the diversity of floral polymorphisms known in *Primula* and provides a valuable system for further exploring the evolutionary history, phylogenetic significance, and relationships between short-styled homostyly and related lineages.

Based on the above scientific questions, this study focused on the putative new short-styled homostylous taxon *Primula panzhouensis* from the karst regions of southwestern China. Using complete cp genome sequences from 16 samples of *Sect. Petiolares*, we conducted comprehensive comparative cp genomic and phylogenetic analyses to address the following objectives: (1) to characterize the structural features and variation patterns of cp genomes in this group and assess its phylogenetic position based on cp genome data; (2) to evaluate the taxonomic status of this putative lineage as a distinct taxonomic entity by integrating morphological evidence, and to document its rare short-styled homostylous floral trait; and (3) to investigate its distribution patterns and potential ecological context by incorporating geographical distribution and habitat characteristics.

By integrating comparative cp genomics, phylogenetic reconstruction, morphological comparisons, and field observations, this study aims to clarify the taxonomic position of the putative new *P. panzhouensis* lineage, evaluate the degree of support for its recognition as an independent taxonomic entity, and provide new insights into floral diversity and its phylogenetic significance within *Primula*.

## 2 Materials and methods

### 2.1 Sample collection

From 2023 to 2026, field investigations and sampling were conducted on one putative new species and several closely related taxa within Sect. *Petiolares*. A total of 16 samples representing five species were collected, encompassing multiple geographic populations. The sampled taxa included putative new species and its closely related species *Primula ovalifolia*, *P. esquirolii*, *P. tardiflora*, and *P. bergenioides*. For each species, several living individuals and fresh leaf samples were collected. Extensive photographs of habitats, as well as detailed images of morphological and anatomical structures, were obtained. Fresh leaves were immediately placed in sealed bags containing silica gel for rapid desiccation and subsequently used for cp genome sequencing. Living plants were transported back for the preparation of voucher specimens and for detailed morphological characterization, including measurement and observation of diagnostic traits. All voucher specimens were deposited in the Institute of Medicinal Plant Cultivation (Breeding) and Processing, Guizhou University of Traditional Chinese Medicine. Detailed information, including collection localities, sample numbers, number of voucher specimens, and the genbank accession is provided in **Table 1**. In addition, the complete cp genome sequences of other related samples used in this study were retrieved from the National Center for Biotechnology Information (NCBI, https://www.ncbi.nlm.nih.gov/), and their corresponding accession numbers are listed in **Table 2**.

**Table 1:** Sample collection information.

ID	Species	Locality	Longitude (E)	Latitude (N)	Voucher no.	GenBank accession
WZK-1	*Primula panzhouensis*	Jichangping Town, Panzhou City, Guizhou Province, China	104.619186°E	25.987501°N	ZKWU20250316001	PZ653550
WZK-2	*Primula ovalifolia*	Shuicheng District, Liupanshui City, Guizhou Province, China	104.729607°E	26.106852°N	ZKWU20250317001	PZ653551
WZK-3	*Primula esquirolii*	Fahe Village, Hezhang County, Bijie City, Guizhou Province, China	104.913055°E	27.057198°N	ZKWU20250318001	PZ653552
WZK-4	*Primula ovalifolia*	Fahe Village, Hezhang County, Bijie City, Guizhou Province, China	104.913071°E	27.057196°N	ZKWU20250318001	PZ653553
WZK-5	*Primula esquirolii*	Jiudongtian Scenic Area, Dafang County, Bijie City, Guizhou Province, China	105.34771°E	26.959597°N	ZKWU20250319001	PZ653554
WZK-6	*Primula esquirolii*	Tianshengqiao, Nayong County, Bijie City, Guizhou Province, China	105.27964°E	26.773321°N	ZKWU20250319002	PZ653555
WZK-7	*Primula esquirolii*	Pingba Tiankeng, Pingba District, Anshun City, Guizhou Province, China	106.240608°E	26.416887°N	ZKWU20250425001	PZ653556
WZK-8	*Primula esquirolii*	No. 74 Guihuang Highway, Pingba District, Anshun City, Guizhou Province, China	106.233231°E	26.403255°N	ZKWU20250425002	PZ653557
WZK-9	*Primula esquirolii*	Qingzhen City, Guiyang City, Guizhou Province, China	106.470715°E	26.556079°N	ZKWU20240206001	PZ653558
WZK-10	*Primula ovalifolia*	Yankantou, Houshan Town, Xuyong County, Luzhou City, Sichuan Province, China	105.33105163°E	27.915476°N	ZKWU20240321001	PZ653559
WZK-11	*Primula ovalifolia*	Yankantou, Houshan Town, Xuyong County, Luzhou City, Sichuan Province, China	105.33105163°E	27.915476°N	ZKWU20240321002	PZ653560
WZK-12	*Primula tardiflora*	Longcanggou Town, Yingjing County, Ya’an City, Sichuan Province, China	102.88907471°E	29.619421°N	ZKWU20240323001	PZ653561
WZK-13	*Primula tardiflora*	Longcanggou Town, Yingjing County, Ya’an City, Sichuan Province, China	102.88907471°E	29.619421°N	ZKWU20240323002	PZ653562
WZK-14	*Primula bergenioides*	Xiling Snow Mountain, Dayi County, Chengdu City, Sichuan Province, China	103.171633°E	30.675123°N	ZKWU20240602001	PZ653563
WZK-15	*Primula ovalifolia*	Hetao Village, Shadaogou Town, Xuan’en County, Enshi Tujia and Miao Autonomous Prefecture, Hubei Province, China	109.570082°E	29.696057°N	ZKWU20240401001	PZ653564
WZK-16	*Primula bergenioides*	Tangba Village, Sanhe Town, Jianyang City, Chengdu City, Sichuan Province, China	104.740036°E	30.499029°N	ZKWU20230307001	PZ653565

**Table 2:** GenBank accession numbers of the chloroplast genomes downloaded from NCBI.

Family	Tribe	Genus	Section	Species	GenBank
Primulaceae	Trib. Primuleae	*Primula*	Sect. *Petiolares*	*Primula moupinensis*	NC 050244.1
				*Primula hookeri*	NC 053593.1
				*Primula sonchifolia*	NC 053594.1
				*Primula strumosa*	NC 053599.1
				*Primula taliensis*	NC 053601.1
				*Primula ovalifolia*	NC 064961.1
				*Primula sinuata*	NC 088046.1
				*Primula meishanensis*	PQ213816.1
				*Primula dejuniana*	PQ213817.1
				*Primula wawushanica*	PQ568378.1
Primulaceae	Trib. Primuleae	*Primula*	Sect. *Denticulata*	*Primula denticulata*	NC 050247.1
				*Primula erythrocarpa*	NC 053598.1
Primulaceae	Trib. Primuleae	*Primula*	Sect. *Aleuritia*	*Primula knuthiana*	NC 039350.1
				*Primula pulchella*	NC 050246.1
				*Primula stenocalyx*	NC 058249.1
				*Primula pumilio*	NC 065465.1
				*Primula farinosa*	PP541897.1
				*Primula fistulosa*	PQ412516.1
				*Primula flava*	PQ536122.1
				*Primula gemmifera*	PQ536123.1
				*Primula fangii*	PQ838449.1
Primulaceae	Trib. Primuleae	*Primula*	Sect. *Auriculastrum*	*Primula palinuri*	NC 061708.1
				*Primula daonensis*	PP541896.1
				*Primula spectabilis*	PP541899.1
Primulaceae	Trib. Primuleae	*Primula*	Sect. *Crystallophlomis*	*Primula calliantha*	MZ054238.1
				*Primula handeliana*	NC 039348.1
				*Primula woodwardii*	NC 039349.1
				*Primula szechuanica*	NC 080275.1
				*Primula farreriana*	PP842194.1
				*Primula purdomii*	PQ516712.1
				*Primula agleniana*	PV866628.1
Primulaceae	Trib. Lysimachieae	*Lysimachia*	Sect. *Apodanthera*	*Lysimachia chapaensis*	PP235257.1
Primulaceae	Trib. Primuleae	*Androsace*	Sect. *Chamaejasme*	*Androsace laxa*	NC 039347.1

### 2.2 Chloroplast genome DNA extraction, library construction, and sequencing

Fresh, young leaves without visible signs of disease or herbivore damage were collected for DNA extraction. Total genomic DNA was extracted using a modified CTAB method (Doyle and Doyle, 1987; Zhou et al., 2019). The concentration and quality of extracted DNA were assessed using an ultra-micro spectrophotometer and a blue-light transilluminator imaging system, respectively, to ensure that the DNA met the requirements for subsequent library construction and high-throughput sequencing. High-throughput sequencing was performed on the Illumina HiSeq X-Ten platform using paired-end libraries (2 × 150 bp). Approximately 10 Gb of raw sequencing data were generated for each sample. Library preparation and sequencing were conducted at the Molecular Biology Experimental Platform of the Kunming Institute of Botany, Chinese Academy of Sciences.

### 2.3 Chloroplast genome assembly and annotation

Raw sequencing reads were initially subjected to quality assessment and filtering using FastQC and Fastp (Chen et al., 2018). FastQC was used to evaluate the overall quality distribution of the raw reads and identify potential sequencing issues, whereas Fastp was employed to remove adapter sequences, low-quality reads, and reads containing excessive ambiguous nucleotides (N). The resulting high-quality clean reads were subsequently used for cp genome assembly. To improve the reliability of cp genome reconstruction, two assembly programs based on distinct algorithmic strategies, GetOrganelle (Jin et al., 2020) and NOVOPlasty (Dierckxsens et al., 2017), were independently applied to assemble the cp genomes of each sample. The assembly outputs generated by the two approaches were compared, and all samples showed identical or highly consistent cp genome structures. For regions showing potential discrepancies between assemblies, raw reads were mapped back to the assembled cp genome sequences and manually inspected and corrected in Geneious (Kearse et al., 2012). The completeness of each assembly was further evaluated by visualizing assembly graphs using Bandage (Wick et al., 2015) to confirm the circularized cp genome structure and the connectivity of inverted repeat (IR) regions. The final cp genome assemblies exhibited an average sequencing depth of approximately 427×. Specific structural regions, including IR boundary variations and regions with differences in intron composition, were further validated through independent assembly comparisons, assembly graph inspection, and read-mapping verification. Chloroplast genome annotation was performed using a combination of PGA (Qu et al., 2019), GeSeq (Tillich et al., 2017), and CPGAVAS2 (Shi et al., 2019). The preliminary annotations were subsequently manually curated in Geneious, with particular attention to gene boundaries, exon-intron organization, and IR junction regions. Finally, circular cp genome maps were generated using OGDRAW (Greiner et al., 2019).

### 2.4 Analysis of repetitive sequences

simple sequence repeats (SSRs) in the cp genome were identified using the online program MISA (Beier et al., 2017). The detection parameters were set as follows: the minimum numbers of repeat units for mono-, di-, tri-, tetra-, penta-, and hexanucleotide motifs were 10, 5, 4, 3, 3, and 3, respectively. The minimum distance allowed between two adjacent SSRs was defined as 100 bp.

### 2.5 Codon usage bias analysis

Based on the cp genome annotation results, protein-coding sequences (CDSs) from all samples were extracted using PhyloSuite (Zhang et al., 2020). Codon usage bias was evaluated using CodonW (Shields and Sharp, 1987), including the calculation of amino acid composition and the relative synonymous codon usage (RSCU) values for each CDS. Based on the obtained RSCU values, heatmaps were generated using TBtools (Chen et al., 2022) to visualize variation patterns in codon usage among different samples.

### 2.6 Analysis of IR boundary structure variation

To compare variations in the IR boundary regions among different samples, the IR junctions of the cp genomes from 16 *Primula* Sect. *Petiolares* samples were visualized and compared using CPJSdraw (Li et al., 2023). Both IR copies within the complete cp genome sequences were retained during the analysis. Particular attention was paid to the positional shifts of adjacent genes, expansions or contractions of IR boundaries, and structural differences among samples at the four junction regions: large single-copy (LSC)/IRb, IRb/small single-copy (SSC), SSC/IRa, and IRa/LSC.

### 2.7 Structural variation analysis of chloroplast genomes

Using the cp genome of *P.* moupinensis as the reference, whole-genome alignment and visualization of 16 samples belonging to Sect. *Petiolares* were conducted with mVISTA software (Frazer et al., 2004) under the Shuffle-LAGAN alignment mode. To assess sequence divergence across different genomic regions, nucleotide diversity was calculated for the LSC, SSC, and IR regions using DnaSP (Rozas et al., 2017). A sliding window analysis was performed with a window length of 600 bp and a step size of 200 bp. Regions exhibiting elevated Pi values were identified as highly variable regions based on variation patterns and were further evaluated for their potential utility in phylogenetic analyses.

### 2.8 Phylogenetic analysis

Phylogenetic relationships among *Primula* taxa were reconstructed based on complete cp genome sequences, with *Lysimachia chapaensis* and *Androsace laxa* selected as outgroups. A total of 47 *Primula* samples were included in the phylogenetic analyses, comprising 10 species from Sect. *Petiolares*, seven species from Sect. *Crystallophlomis*, nine species from Sect. *Proliferae*, two species from Sect. *Diphyomene*, and three species from Sect. *Auriculastrum* (**Table 2**), together with 16 newly sequenced samples generated in this study. Phylogenetic reconstruction was performed using both maximum likelihood (ML) and bayesian inference (BI) approaches. All complete cp genome sequences were aligned using MAFFT (Katoh et al., 2002), and poorly aligned regions were removed using trimAl under the relaxed mode to minimize the influence of ambiguous alignment regions on phylogenetic inference. The final cp genome alignment matrix consisted of 159,351 bp, including 24,274 variable sites and 12,391 parsimony-informative sites. Because complete cp genomes contain two inverted repeat regions (IRa and IRb), the entire cp genome sequences were retained for phylogenetic analyses without removing or merging the IR regions.

ML analysis was conducted using IQ-TREE (Nguyen et al., 2015). The best-fitting nucleotide substitution model was selected using the ModelFinder Plus function implemented in IQ-TREE based on the Bayesian information criterion (BIC). The TVM+F+R5 model was identified as the optimal model for the ML analysis. The ML phylogenetic tree was reconstructed under this model, and branch support values were assessed using 1,000 ultrafast bootstrap replicates.

BI analysis was performed using MrBayes v3.2.7 (Huelsenbeck and Ronquist, 2001). According to the BIC criterion, ModelFinder selected TVM+F+I+G4 as the best-fitting model for the Bayesian analysis. However, because the TVM (transversion model) is not directly implemented in MrBayes, the GTR model was adopted as an approximation. The GTR model represents a general time-reversible substitution model, whereas the TVM model can be considered a constrained form of the GTR model. Therefore, the GTR+F+I+G4 model provides a more generalized parameter space that can adequately approximate the substitution process described by the TVM+F+I+G4 model. In MrBayes, the GTR substitution model was implemented using lset nst=6, whereas among-site rate variation was modeled using a combination of invariant sites and gamma-distributed rates (lset rates=invgamma ngammacat=4). Empirical base frequencies were fixed using prset statefreqpr=fixed(empirical).

The Bayesian analysis was performed with four Markov chain Monte Carlo (MCMC) chains run simultaneously for 2,000,000 generations, with trees sampled every 1,000 generations. The first 25% of sampled trees were discarded as burn-in, and the remaining trees were used to construct a 50% majority-rule consensus tree with posterior probability (PP) values calculated for each node. The ML and BI phylogenetic trees were visualized and edited using the online tool iTOL (Letunic and Bork, 2021).

### 2.9 Morphological comparison and taxonomic assessment

In order to clarify the morphological differences between the putative new species *P. panzhouensis* and its closely related taxa, we conducted a comprehensive comparative morphological analysis based on continuous field observations, measurements of living plants, and herbarium specimen examinations from major herbaria in China and abroad. For the morphological assessment of the putative new species *P. panzhouensis*, 15 individuals from the same population were selected and repeatedly measured over a four-year period (2023-2026) using a digital vernier caliper to evaluate the stability and variation of diagnostic traits. For each individual, three to five mature flowers and three to five fully developed leaves were randomly selected for measurement. The following morphological characters were recorded: leaf length, leaf width, scape height, number of inflorescences, corolla diameter, corolla tube length, calyx length, style position, and other taxonomically informative traits. In addition, detailed observations of floral structures were performed, with particular emphasis on heterostyly, corolla morphology, and floral dimensional characteristics.

For the closely related taxa, during field sampling, 10 individuals were randomly selected from each population for morphological measurements (including five long-styled and five short-styled individuals for distylous species). The measured morphological traits included leaf length, leaf width, scape height, number of inflorescences, corolla diameter, corolla tube length, calyx length, and style position. In addition, digital specimen resources from six herbaria, including PE, K, P, E, IBSC, and BM, were examined. For species represented by more than 20 specimens, 20 specimens were selected for examination, whereas all available specimens were examined for species with fewer than 20 specimens. A total of 34 herbarium specimens were examined, including 20 specimens of *P. ovalifolia*, 3 specimens of *P. esquirolii*, 7 specimens of *P. tardiflora*, and 4 specimens of *P. bergenioides*. Combined with relevant taxonomic literature (Hu, 1990; Hu and Kelso, 1996), morphological characters of taxonomic significance, particularly floral morph type and floral dimensions, were carefully recorded. Based on these data, the conservation status of the new species was assessed according to the IUCN Red List Categories and Criteria (IUCN Standards and Petitions Committee, 2024). A morphological comparison table and corresponding comparative illustrations were subsequently prepared to facilitate discrimination between the new species and its closely related taxa.

## 3 Results

### 3.1 Characteristics of the chloroplast genomes

The results indicated that all samples displayed the typical circular quadripartite structure characteristic of angiosperm cp genomes. (**Figure 1**; see **Supplementary Figure S1** for the remaining samples). The complete cp genome sizes ranged from 151,076 bp to 153,420 bp. Among them, WZK-9 *P. esquirolii* possessed the largest genome (153,420 bp), followed by WZK-12 *P. tardiflora* and WZK-13 *P. tardiflora*, with lengths of 152,028 bp and 152,068 bp, respectively. The remaining 13 samples all had genome sizes of approximately 151 kb. Each cp genome comprised a LSC region, a SSC region, and a pair of IR regions (IRa and IRb). The length of the LSC region ranged from 82,660 bp to 83,879 bp. In the putative new species WZK-1 *P. panzhouensis*, the LSC region was 82,660 bp in length, whereas in the remaining 15 samples it was approximately 83 kb. The length of the SSC region ranged from 16,369 bp to 17,815 bp. In WZK-9 *P. esquirolii*, the SSC region was 16,369 bp, whereas in the remaining 15 samples it was approximately 17 kb. The IR region ranged from 25,135 bp to 26,917 bp in length; in WZK-9 *P. esquirolii* it was 26,917 bp, while in the other 15 samples it was approximately 25 kb. The total GC content of the putative new species and the 15 samples of Sect. *Petiolares* showed only slight variation, ranging from 36.97% to 37.05%. However, marked differences in GC content were observed among different genomic regions. The IR regions exhibited the highest GC content (42.07%-42.95%), which was significantly higher than that of the LSC region (34.88%-34.95%) and the SSC region (30.08%-30.48%). This pattern is consistent with the typical distribution of GC content in cp genomes of higher plants. The total number of genes encoded in the 16 cp genomes ranged from 131 to 133, comprising 86–88 protein-coding genes, 37 tRNA genes, and 8 rRNA genes (**Table 3**).

**Figure 1:**
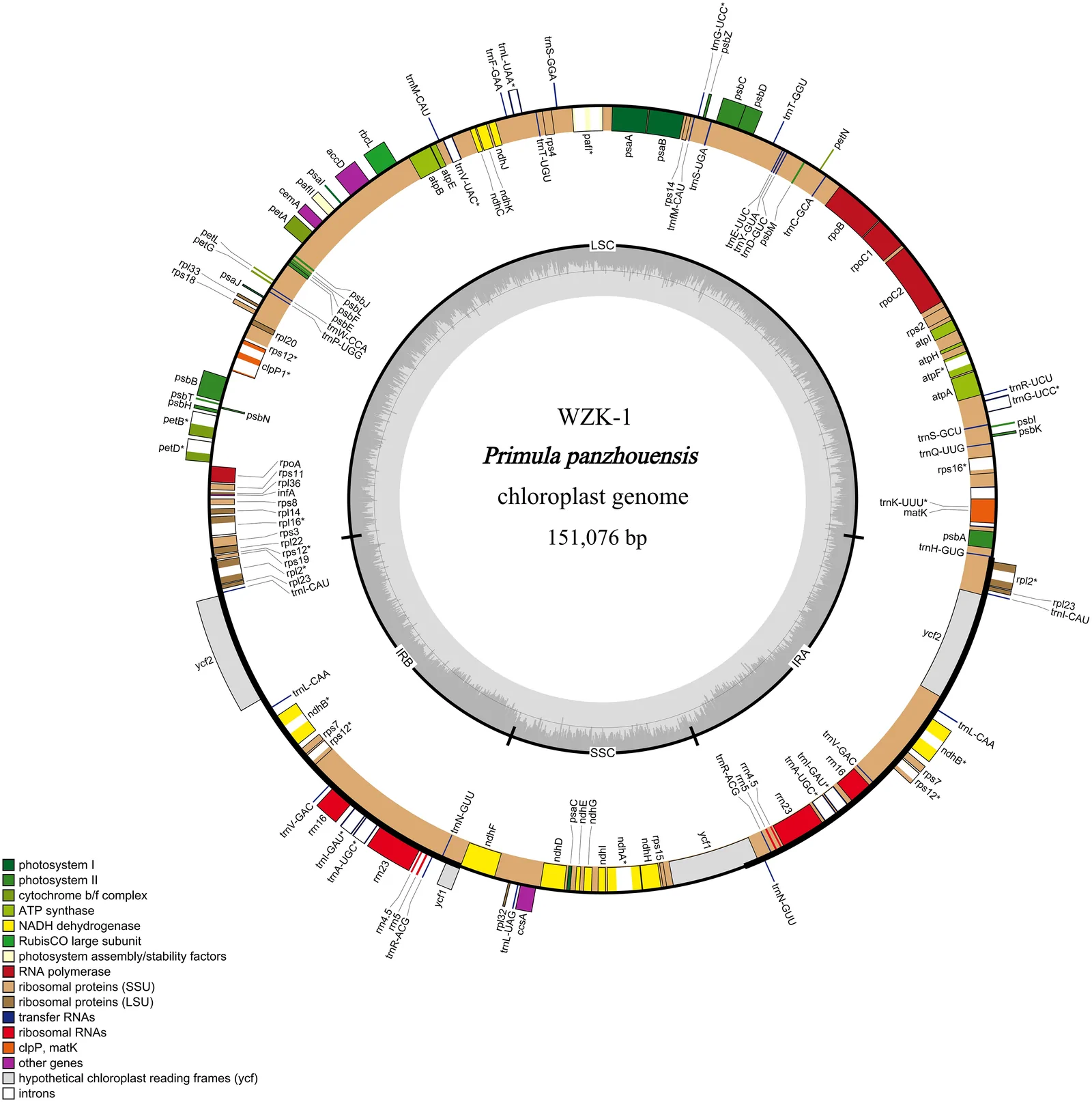
Complete chloroplast genome map of WZK-1 *Primula panzhouensis*.

**Table 3:** Chloroplast genome features of 16 samples.

Species	Total genome			Large single-copy (LSC) region			Small single-copy (SSC) region			Inverted repeat (IR) regions		Total genes	Protein-coding genes	rRNA genes	tRNA genes
	Length (bp)	GC content (%)	Length (bp)		GC content (%)	Length (bp)		GC content (%)	Length (bp)		GC content (%)				
WZK-1 *Primula panzhouensis*	151076	37.05	82660		34.93	17738		30.22	25339		42.89	131	86	8	37
WZK-2 *Primula ovalifolia*	151527	37.02	83366		34.91	17789		30.11	25186		42.95	131	86	8	37
WZK-3 *Primula esquiroli*i	151654	37.04	83518		34.91	17708		30.25	25214		42.93	133	88	8	37
WZK-4 *Primula ovalifoli*a	151352	37.03	83290		34.91	17676		30.21	25193		42.92	131	86	8	37
WZK-5 *Primula esquirolii*	151720	37.03	83535		34.94	17815		30.16	25185		42.95	133	88	8	37
WZK-6 *Primula esquirolii*	151761	37.03	83609		34.91	17760		30.22	25196		42.94	133	88	8	37
WZK-7 *Primula esquirolii*	151712	37.05	83547		34.94	17751		30.23	25207		42.94	133	88	8	37
WZK-8 *Primula esquirolii*	151678	37.05	83533		34.94	17749		30.23	25198		42.95	133	88	8	37
WZK-9 *Primula esquirolii*	153420	36.97	83217		34.95	16369		30.48	26917		42.07	133	88	8	37
WZK-10 *Primula ovalifolia*	151556	37.02	83384		34.92	17796		30.11	25188		42.94	131	86	8	37
WZK-11 *Primula ovalifolia*	151551	37.03	83378		34.93	17797		30.11	25188		42.94	131	86	8	37
WZK-12 *Primula tardiflora*	152028	37.01	83879		34.91	17785		30.22	25182		42.92	133	88	8	37
WZK-13 *Primula tardiflora*	152068	37.02	83871		34.91	17785		30.22	25206		42.93	133	88	8	37
WZK-14 *Primula bergenioides*	151829	36.99	83835		34.88	17724		30.08	25135		42.93	133	88	8	37
WZK-15 *Primula ovalifolia*	151576	37.01	83395		34.9	17807		30.09	25187		42.94	131	86	8	37
WZK-16 *Primula bergenioides*	151912	37	83823		34.89	17723		30.08	25183		42.95	133	88	8	37

The results of functional annotation indicated that the shared annotated genes of the cp genomes of one putative new species and 15 samples of Sect. *Petiolares* be classified into five major categories: photosynthesis, genetic system, biosynthesis and metabolism, conserved open reading frames, and other genes. Further analyses revealed that a total of 15 genes contained a single intron, namely *petB*, *petD*, *atpF*, *ndhA*, *ndhB* (×2), *rpl16*, *rpl2* (×2), *rps16*, *trnK-UUU*, *trnG-UCC* (×2, of which only one copy contains an intron), *trnL-UAA*, *trnV-UAC*, *trnI-GAU* (×2), and *trnA-UGC* (×2). The *rpoC1* gene contained an intron in 15 of the analyzed samples, whereas this intron was absent in the WZK-1 *P. panzhouensis* cp genome. In addition, four genes possessed two introns, including *rps12* (×2), *clpP1*, and *pafI*. Moreover, a total of 19 genes were present in two copies, namely *ndhB*, *rpl2*, *rpl23*, *rps12*, *rps7*, *rrn16*, *rrn23*, *rrn4.5*, *rrn5*, *trnG-UCC*, *trnI-CAU*, *trnL-CAA*, *trnV-GAC*, *trnI-GAU*, *trnA-UGC*, *trnR-ACG*, *trnN-GUU*, *ycf1*, and *ycf2* (**Table 4**). Additionally, *rps12* is a typical trans-spliced gene, with its 5’-end coding region located in the LSC region and its 3’-end coding region situated in the IR region.

**Table 4:** Classification of annotated genes shared by the chloroplast genomes of 16 samples.

Gene function	Group of genes	Name of genes
Photosynthesis	Subunits of photosystem I	*psaA*, *psaB*, *psaC*, *psaI*, *psaJ*
	Subunits of photosystem II	*psbA*, *psbB*, *psbC*, *psbD*, *psbE*, *psbF*, *psbH*, *psbI*, *psbJ*, *psbK*, *psbL*, *psbM*, *psbN*, *psbT*, *psbZ*
	Subunits of cytochrome b/f complex	*petA*, *petB^a^*, *petD^a^*, *petG*, *petL*, *petN*
	Subunits of ATP synthase	*atpA*, *atpB*, *atpE*, *atpF^a^*, *atpH*, *atpI*
	Subunits of NADH-dehydrogenase	*ndhA^a^*, *ndhB^a^* (×2), *ndhC*, *ndhD*, *ndhE*, *ndhF*, *ndhG*, *ndhH*, *ndhI*, *ndhJ*, *ndhK*
	Subunit of rubisco	*rbcL*
Genetic system	Large subunit of ribosome	*rpl14*, *rpl16^a^*, *rpl2^a^* (×2), *rpl20*, *rpl22*, *rpl23* (×2), *rpl32*, *rpl33*, *rpl36*
	Small subunit of ribosome	*rps11*, *rps12^b^* (×2), *rps14*, *rps15*, *rps16^a^*, *rps18*, *rps19*, *rps2*, *rps3*, *rps4*, *rps7* (×2), *rps8*
	rRNA genes	*rrn16* (×2), *rrn23* (×2), *rrn4.5* (×2), *rrn5* (×2)
	tRNA genes	*trnH-GUG*, *trnK-UUU^a^*, *trnQ-UUG*, *trnS-GCU*, *trnG-UCC^a^* (×2), *trnR-UCU*, *trnC-GCA*, *trnD-GUC*, *trnY-GUA*, *trnE-UUC*, *trnT-GGU*, *trnS-UGA*, *trnfM-CAU*, *trnS-GGA*, *trnT-UGU*, *trnL-UAA^a^*, *trnF-GAA*, *trnV-UAC^a^*, *trnM-CAU*, *trnW-CCA*, *trnP-UGG*, *trnI-CAU* (×2), *trnL-CAA* (×2), *trnV-GAC* (×2), *trnI-GAU^a^* (×2), *trnA-UGC^a^* (×2), *trnR-ACG* (×2), *trnN-GUU* (×2), *trnL-UAG*
	DNA dependent RNA polymerase	*rpoA*, *rpoB*, *rpoC1*, *rpoC2*
	Maturase	*matK*
	Translational initiation factor	*infA*
Biosynthesis and metabolism	Subunit of Acetyl-CoA-carboxylase	*accD*
	Protease	*None*
	c-type cytochrom synthesis gene	*ccsA*
	Envelop membrane protein	*cemA*
Conserved ORFs	Conserved open reading frames	*ycf1* (×2), *ycf2* (×2)
Other Genes	Other genes	*clpP1^b^*, *pafI^b^*, *pafII*

^a^genes with one intron; ^b^genes with two introns; ×2, two copies.

### 3.2 Analysis of simple sequence repeats

A total of 587 SSR loci were identified across the 16 samples in this study. Based on the length of the repeat motifs, these SSRs were classified into five types: mononucleotide, dinucleotide, trinucleotide, tetranucleotide, and pentanucleotide repeats (**Figure 2**). Among the identified SSR loci, mononucleotide repeats were the most abundant, accounting for 69.68% of the total SSRs, followed by dinucleotide repeats (17.55%). Tetranucleotide and trinucleotide repeats represented 7.84% and 4.09% of all SSR loci, respectively, whereas pentanucleotide repeats were the least frequent, comprising only 0.85%.

**Figure 2:**
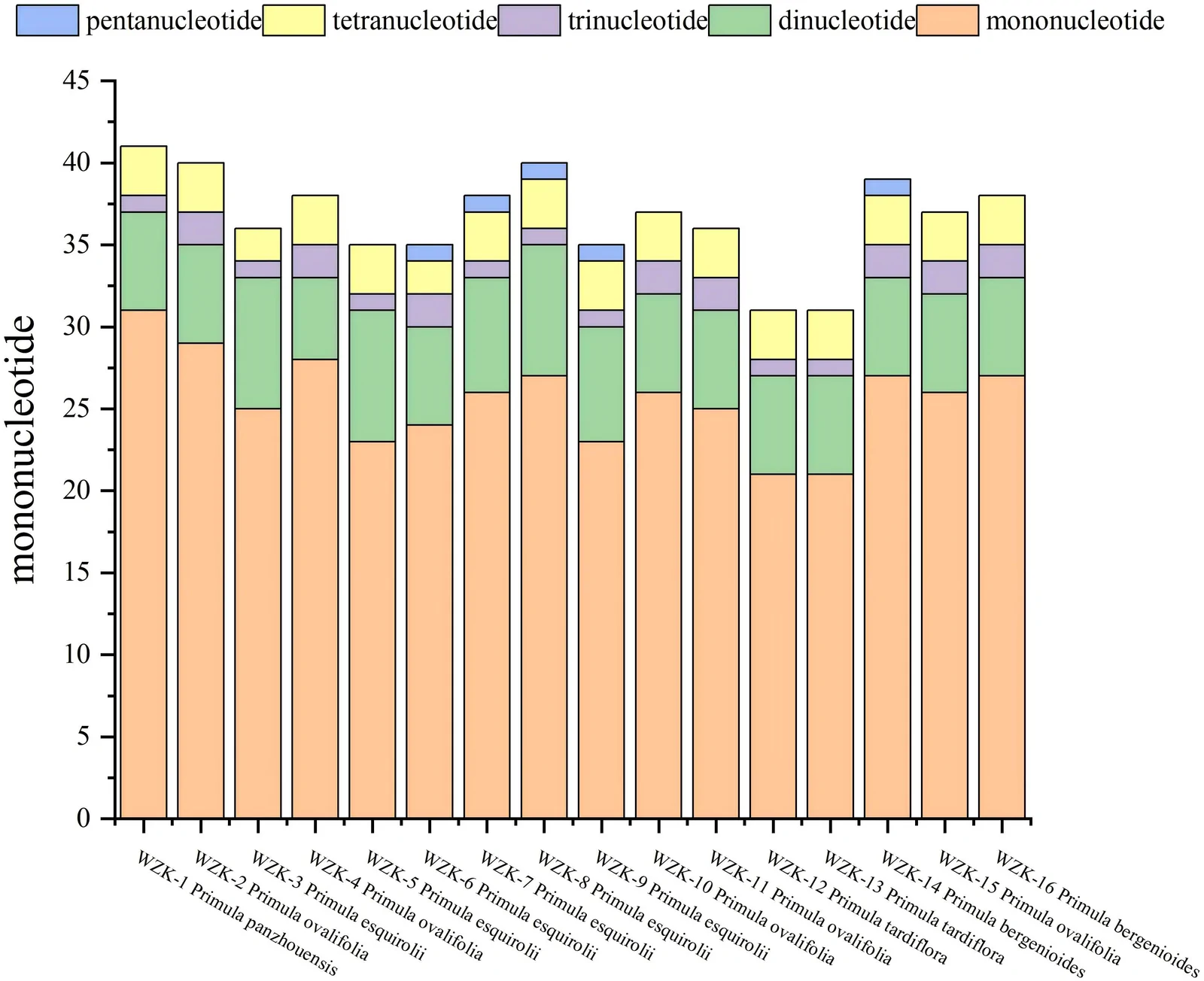
Statistics of the total number of simple sequence repeats (SSRs) in the chloroplast genomes of 16 samples.

Regarding the composition of repeat motifs, a total of 4,334 A/T motifs and only 82 C/G motifs were identified among the mononucleotide repeats, with A/T motifs accounting for 98.14% of all mononucleotide repeats (**Figure 3**). For dinucleotide repeats, only the AT/TA motif was detected, with a total of 567 loci. Three types of trinucleotide repeat motifs were identified, including ATA/TAT, ATT, and AAT/TTA. Among them, the ATA/TAT motif was the most abundant, with 80 loci detected, followed by the ATT motif with 20 loci, whereas the AAT/TTA motif was the least frequent, with only 12 loci identified. A total of four tetranucleotide repeat motifs were detected, namely AAAG, AAAT/TTTA, AATA/TATT, and TTAT. The AATA/TATT motif was predominant, with 96 loci identified, followed by the AAAT/TTTA motif (30 loci), the AAAG motif (9 loci), and the TTAT motif, which exhibited the lowest frequency with only three loci. For pentanucleotide repeats, two motif types, AAAAG and TTTAT, were identified, with 15 loci detected in total. Among these, the TTTAT motif accounted for 12 loci, whereas the AAAAG motif occurred only three times.

**Figure 3:**
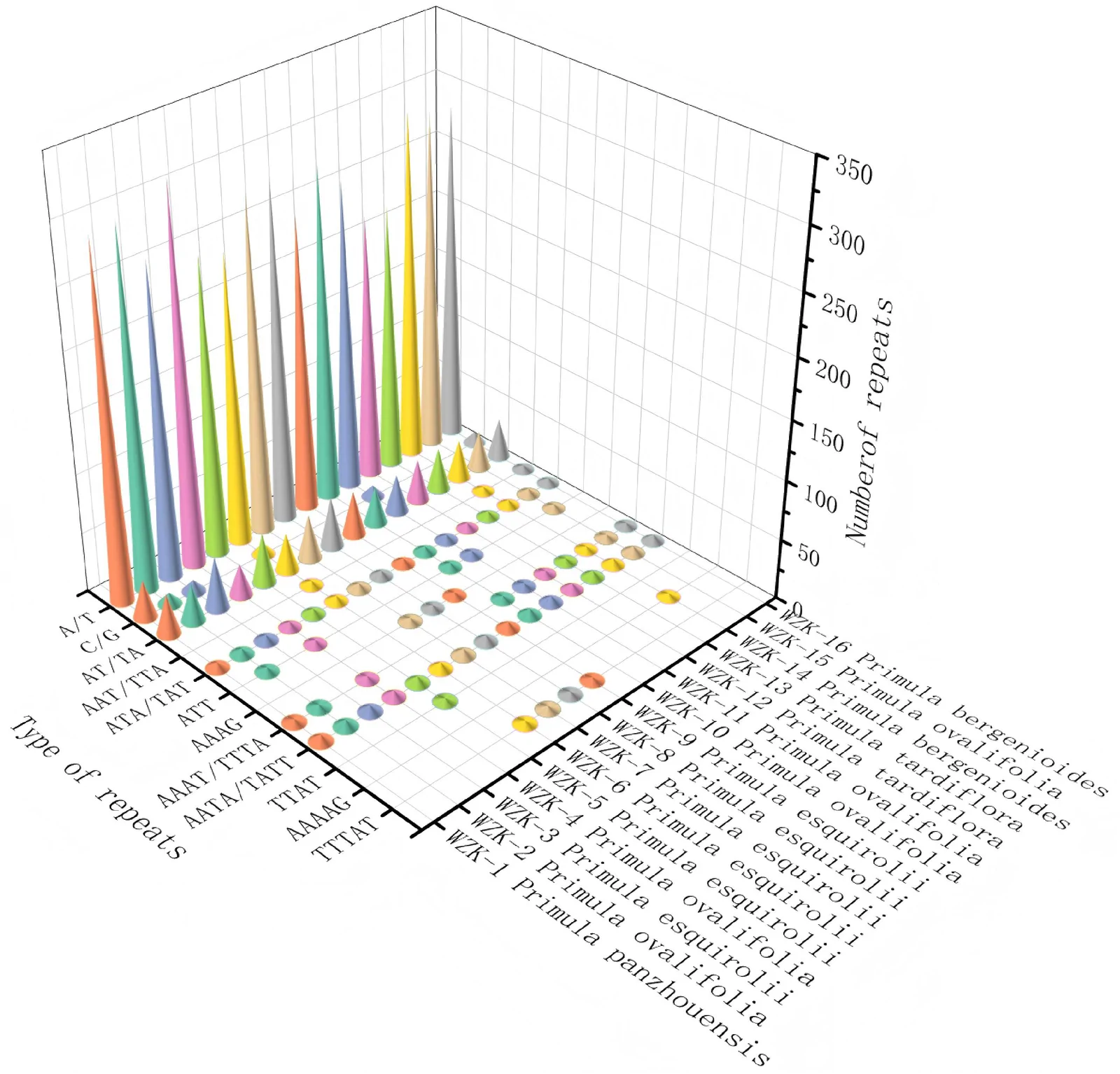
SSRs in the chloroplast genomes of 16 samples.

The total number of SSR loci exhibited a certain degree of variation among the 16 samples. The highest number of SSR loci was detected in WZK-1 *P. panzhouensis* (41 loci), whereas WZK-5 *P. esquirolii*, WZK-6 *P. esquirolii*, and WZK-9 *P. esquirolii* showed the lowest numbers (35 loci each). These results indicate that the SSR composition of the cp genomes in the 16 samples was predominantly characterized by mononucleotide A/T motifs. Although the distribution patterns of different SSR types were generally consistent among samples, variations in SSR abundance were observed.

Among the 16 cp genomes analyzed in this study, WZK-1 *P. panzhouensis* exhibited a relatively higher number of SSR loci. However, because only a single sample of *P. panzhouensis* was included in the present study, whether this difference reflects a species-level characteristic requires further validation through expanded sampling.

### 3.3 Codon usage bias analysis

Codon composition analysis was performed based on the CDSs of the cp genomes from the 16 samples. The amino acid composition analysis revealed that leucine (Leu) was the most abundant amino acid, accounting for 10.11%-11.03% of the total amino acid composition, followed by serine (Ser) and isoleucine (Ile), with proportions ranging from 9.31%-10.24% and 8.73%-9.51%, respectively. In contrast, tryptophan (Trp) and cysteine (Cys) were the least abundant amino acids, accounting for only 1.31%-1.52% and 2.22%-2.46%, respectively. The overall amino acid composition was highly conserved among the different samples. However, Ser, Leu, and Ile exhibited relatively greater variation, with ranges of 0.93%, 0.92%, and 0.78%, respectively. Conversely, Trp and Cys showed the lowest levels of variation, with ranges of only 0.21% and 0.24%, respectively (**Figure 4**).

**Figure 4:**
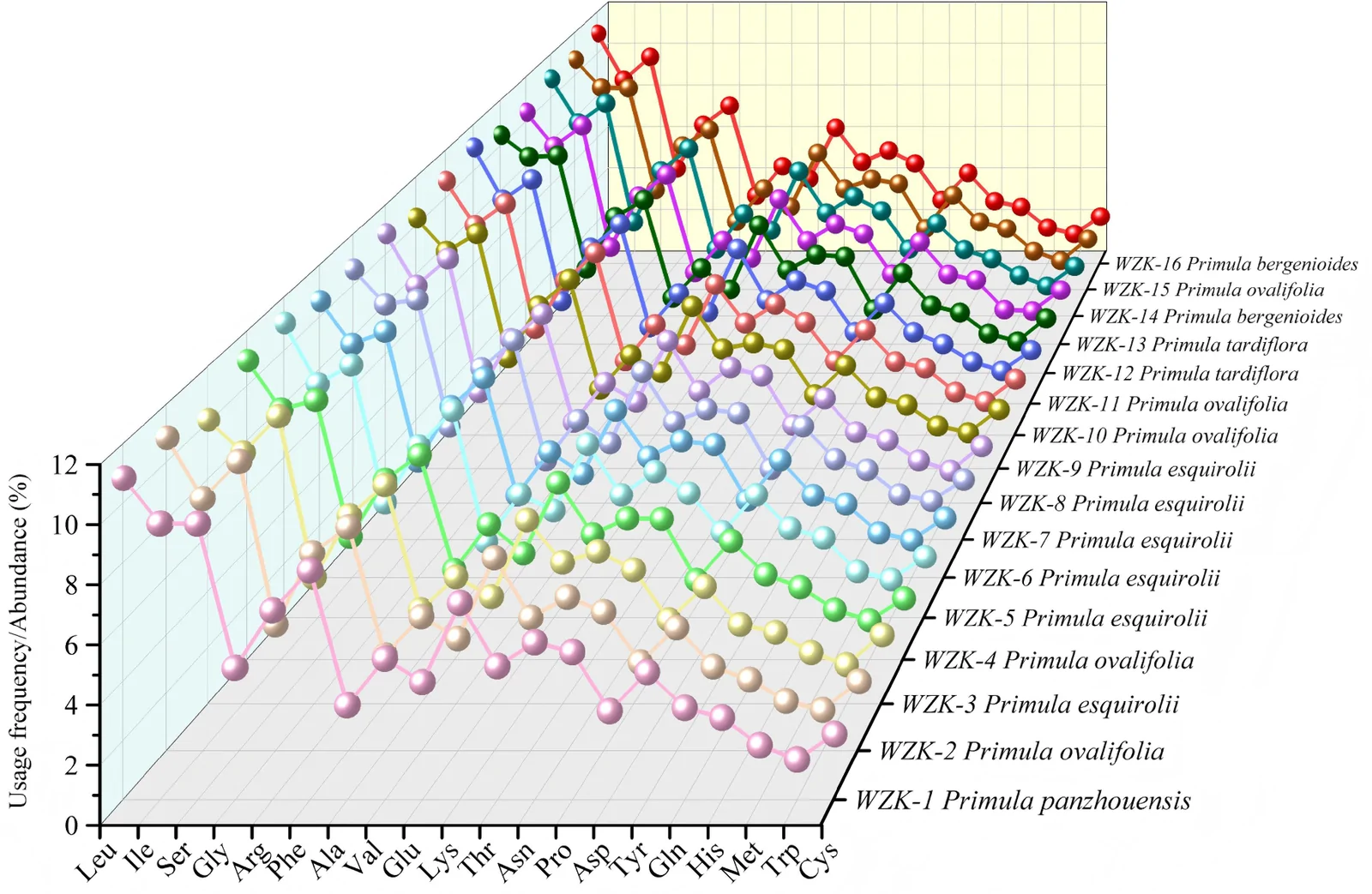
Frequencies of amino acid usage among the 16 samples.

Codon composition analysis revealed that, except for methionine (Met) and Trp, the remaining 18 amino acids were encoded by multiple synonymous codons. Among them, arginine (Arg), Leu, and Ser each possessed six synonymous codons; glycine (Gly), valine (Val), threonine (Thr), proline (Pro), and alanine (Ala) each had four synonymous codons; Ile was encoded by three synonymous codons, whereas the other amino acids were represented by two synonymous codons (**Figure 5**).

**Figure 5:**
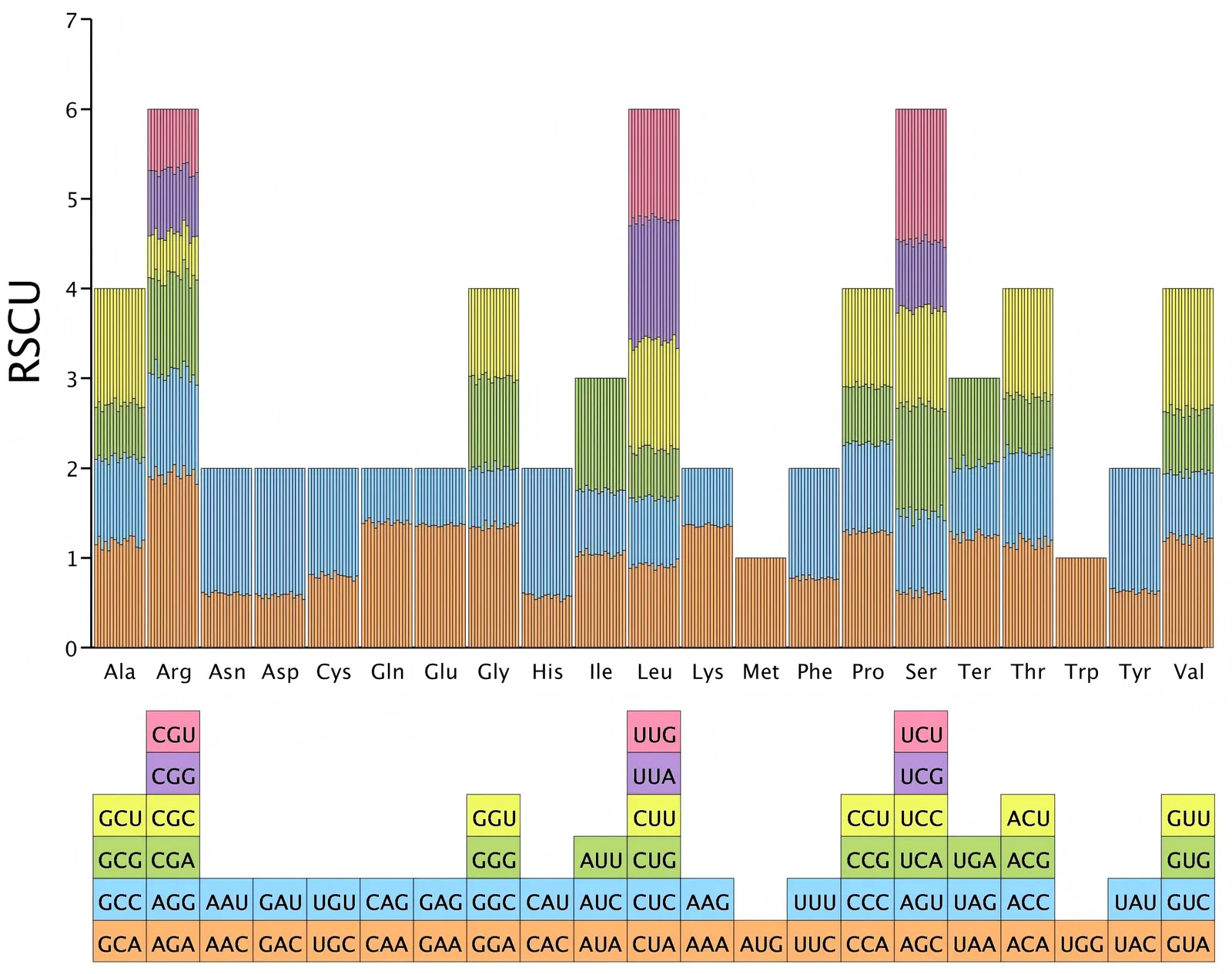
Relative synonymous codon usage (RSCU) of amino acids in the 16 samples.

The RSCU analysis showed that 33 codons exhibited RSCU values greater than 1.00 across the 16 samples, indicating the presence of a certain degree of codon usage bias in the cp genomes analyzed in this study. In contrast, 31 codons showed RSCU values lower than 1.00, representing less frequently used codons. Additionally, the RSCU values of UUG and AUG were both 1.00, suggesting that these codons did not exhibit an obvious preference among synonymous codons (**Figure 6**).

**Figure 6:**
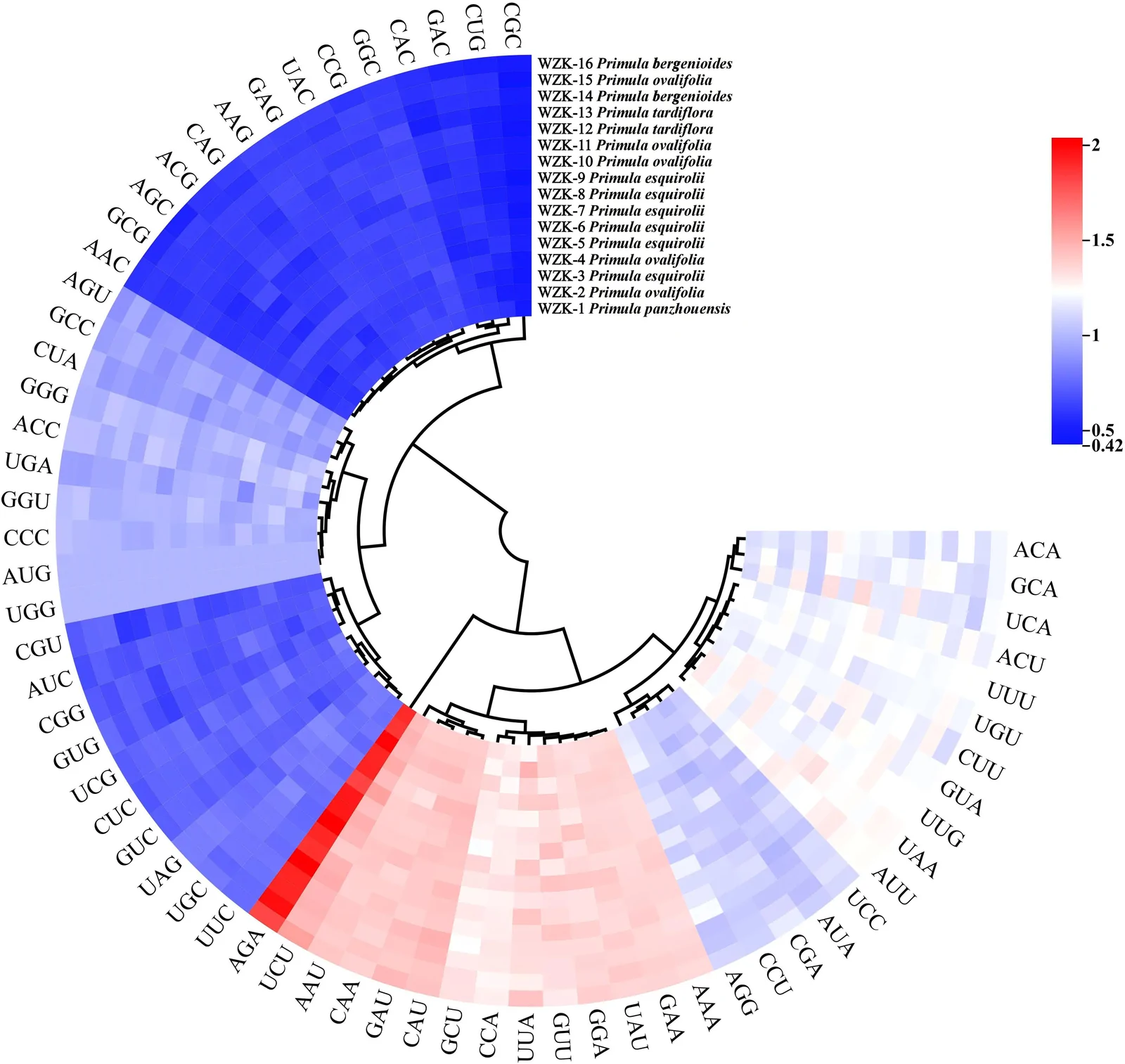
Heatmap of RSCU analysis among the 16 samples.

### 3.4 Contraction and expansion of the IR regions

To investigate structural variations in the cp genomes of the 16 samples, comparative analyses of the IR boundaries were conducted (**Figure 7**). The results revealed that the total length of the IR regions varied among the sampled individuals, ranging from 25,135 bp to 26,917 bp. Structural variations of different degrees were detected at all four junction regions, including LSC/IRb, IRb/SSC, SSC/IRa, and IRa/LSC.

**Figure 7:**
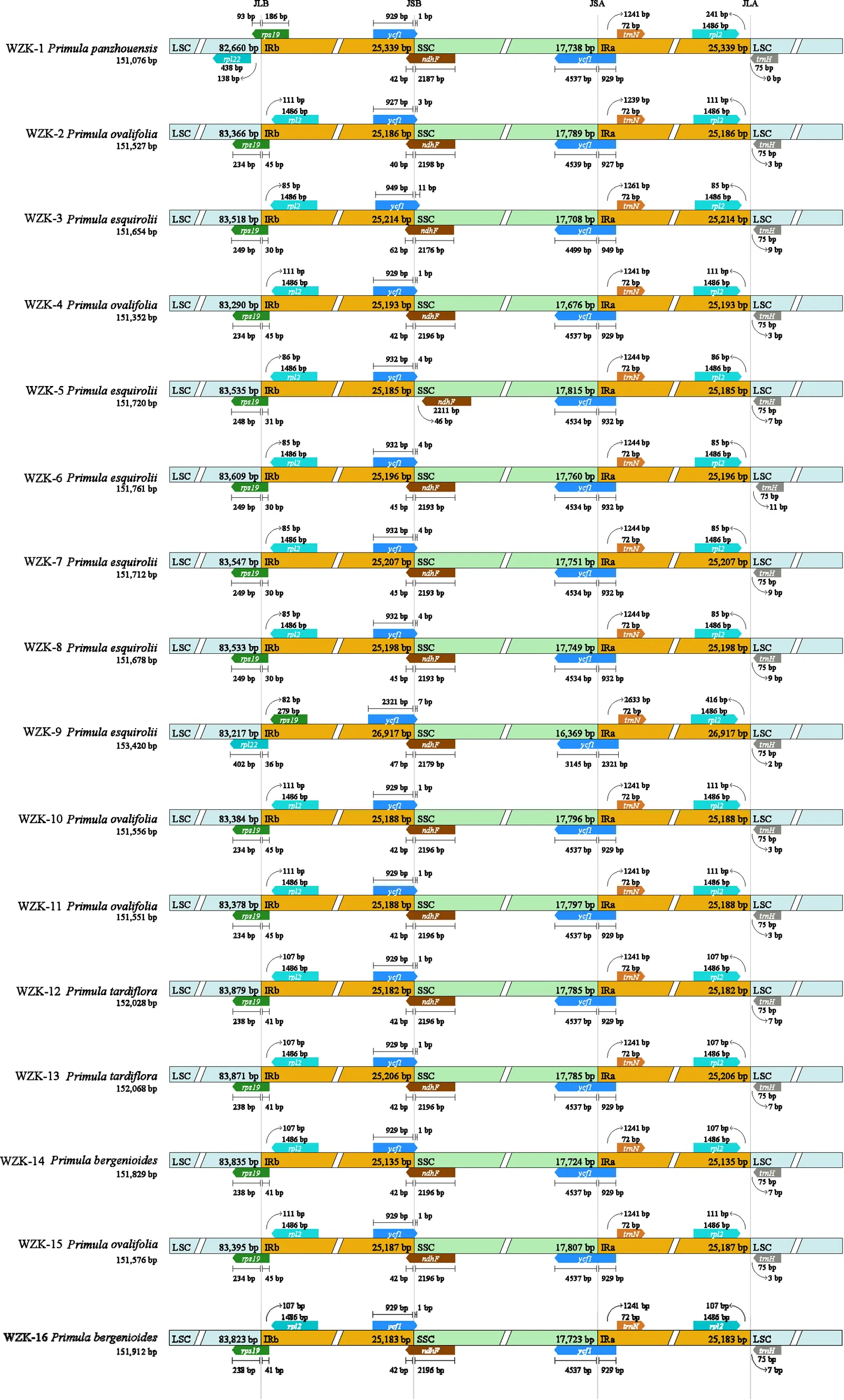
Comparison of the boundary regions among the four partitions of the chloroplast genomes in the 16 samples.

At the LSC/IRb boundary, the positional relationships between the boundary and three genes, namely *rps19*, *rpl22*, and *rpl2*, exhibited variations among the sampled individuals. The *rps19* gene (279 bp) crossed the LSC/IRb boundary in most sampled individuals, with its 3′ end located in the LSC region and positioned 234–249 bp away from the boundary, whereas its 5′ end extended 30–45 bp into the IRb region. However, in WZK-1 *P. panzhouensis*, the 5′ end of *rps19* was located 186 bp from the boundary, with the 3′ end extending 93 bp into the LSC region. In WZK-9 *P. esquirolii*, *rps19* was completely located within the IRb region, with its nearest distance to the boundary being 82 bp. The *rpl22* gene (438 bp) was completely located within the LSC region in most sampled individuals, whereas it crossed the LSC/IRb boundary in WZK-9 *P. esquirolii*, with its 5′ end extending 36 bp into the IRb region. The *rpl2* gene (1,486 bp) was entirely located within the IRb region in all sampled individuals; however, the distances between *rpl2* and the LSC/IRb boundary in WZK-1 *P. panzhouensis* and WZK-9 *P. esquirolii* were considerably greater (241 bp and 416 bp, respectively) than those observed in the other sampled individuals (85–111 bp). The above results indicate that WZK-1 *P. panzhouensis* and WZK-9 *P. esquirolii* exhibited structural variations at the LSC/IRb junction, characterized by a relative expansion of the IRb region.

At the IRb/SSC boundary, the *ycf1* gene crossed the junction, with the majority of its 5′ end located within the IRb region, extending 927–949 bp from the boundary, while its 3′ end extended only 1–11 bp into the SSC region. However, in WZK-9 *P. esquirolii*, the distance between the 5′ end of *ycf1* and the IRb/SSC boundary reached 2,321 bp, indicating a substantial expansion of the IRb region toward the SSC region in this individual. The *ndhF* gene crossed the IRb/SSC boundary in all sampled individuals except WZK-5 *P. esquirolii*, in which it was completely located within the SSC region. In the remaining individuals, the 3′ end of *ndhF* extended 40–62 bp into the IRb region. At the SSC/IRa boundary, the *ycf1* gene also crossed the boundary, with its 5′ end located within the IRa region and positioned 927–949 bp from the boundary (2,321 bp in WZK-9 *P. esquirolii*), whereas its 3′ end was located within the SSC region. The distance between the 3′ end of *ycf1* and the SSC/IRa boundary varied considerably among the sampled individuals, ranging from 3,145 bp to 17,815 bp, which was mainly attributed to differences in the extent of IR expansion toward the SSC region. The *trnN* gene (72 bp) was completely located within the IRa region, with its distance from the SSC/IRa boundary ranging from 1,239 bp to 1,261 bp, except for WZK-9 *P. esquirolii*, in which the distance reached 2,633 bp. At the IRa/LSC boundary, the *trnH* gene (75 bp) was completely located within the LSC region in all sampled individuals, with its distance from the boundary ranging from 0 to 11 bp. The *rpl2* gene was entirely located within the IRa region, and its distance from the IRa/LSC boundary in WZK-1 *P. panzhouensis* and WZK-9 *P. esquirolii* was considerably greater (241 bp and 416 bp, respectively) than that observed in the other sampled individuals (85–111 bp). These results further indicate that WZK-1 *P. panzhouensis* and WZK-9 *P. esquirolii* exhibited remarkable IR expansion at multiple junction regions, particularly involving the IRb and IRa regions.

The above results indicate that WZK-1 *P. panzhouensis* and WZK-9 *P. esquirolii* exhibited structural variations associated with shifts in the LSC/IRb boundary position and relative expansion of the IRb region. It should be noted that, as cp genome data are currently available for only a single individual of *P. panzhouensis*, the observed boundary characteristics (e.g., IRb expansion) should be considered as observations based on this individual sample. Whether these features represent stable species-level characteristics of *P. panzhouensis* requires further validation using additional individuals.

### 3.5 Sequence polymorphism analysis

Using the cp genome of *P. moupinensis* as the reference, comparative cp genomic analysis of the 16 samples was conducted using mVISTA. The results revealed that the cp genomes of these samples were highly conserved in overall structure. The coding regions exhibited relatively high sequence conservation, although variations were detected in the coding regions of several genes, including *rps8*, *rpl22*, *rps19*, and *ycf2*. In contrast, the non-coding regions showed substantially higher levels of sequence variation than the coding regions. The major mutation hotspots were primarily distributed in intergenic spacer (IGS) regions, including *trnH-GUG-psbA*, *trnK-UUU-rps16*, *trnG-UCC-trnR-UCU*, *petN-psbM*, *trnD-GUC-trnY-GUA*, *trnT-GGU-psbD*, *trnfM-CAU-rps14*, *trnT-UGU-trnL-UAA*, *trnF-GAA-ndhJ*, *accD-psaI*, *petA-psbJ*, *psbE-petL*, *psaJ-rpl33*, *rpl32-ccsA*, *rps18-rpl20*, *petD-rps11*, *rpl36-rps8*, and *trnI-CAU-ycf2* (**Figure 8**). These highly variable regions may serve as potential candidate regions for the development of molecular markers in future studies of related taxa within Sect. *Petiolares*.

**Figure 8:**
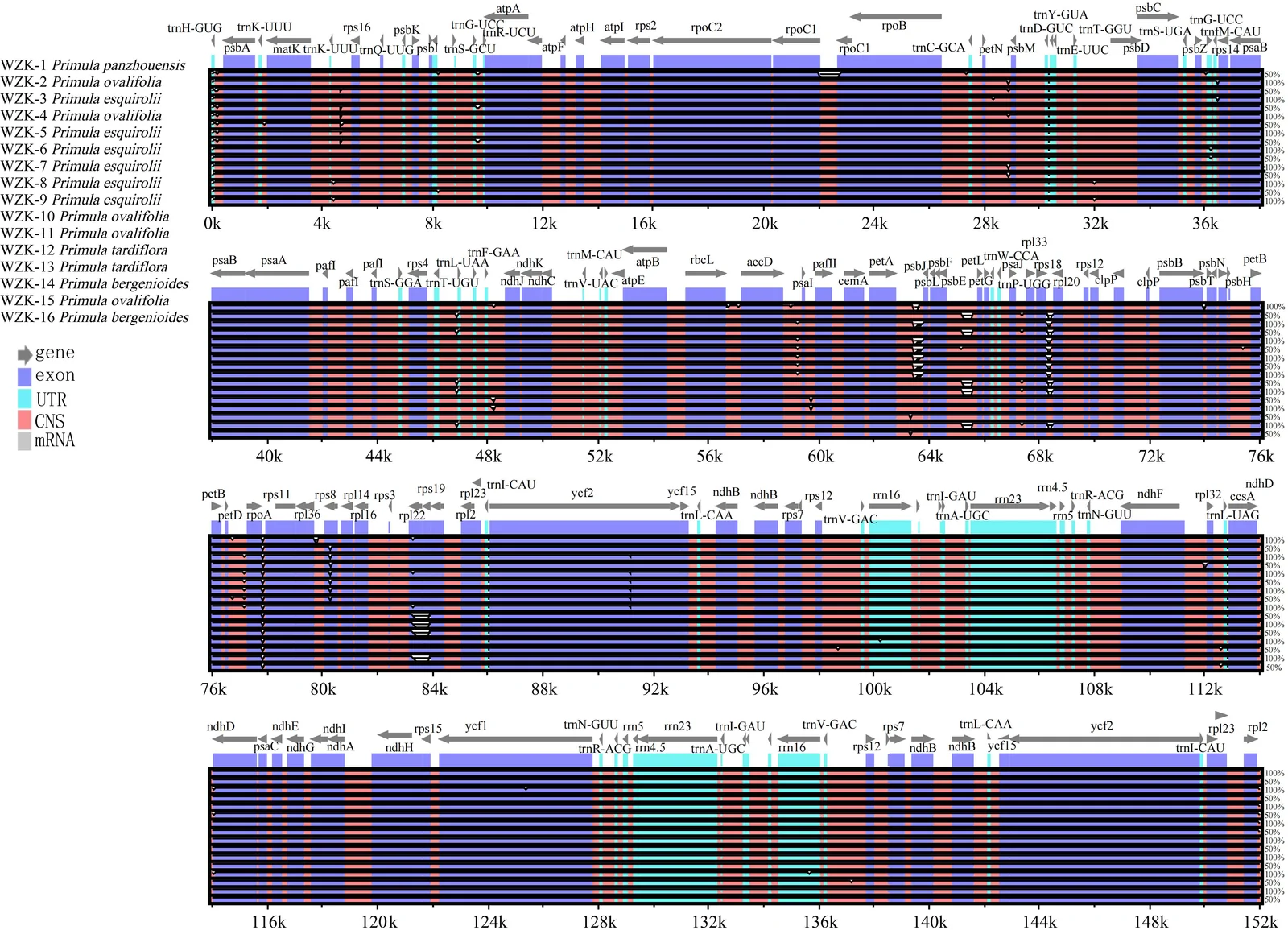
Global alignment of the chloroplast genomes from 16 samples.

To further evaluate the sequence variation among the cp genomes of the 16 samples analyzed in this study, nucleotide diversity (Pi) was calculated using a sliding-window approach based on the multispecies alignment of complete cp genome sequences. The results obtained using DnaSP revealed that the genome-wide Pi values ranged from 0 to 0.02415. Overall, nucleotide diversity exhibited a characteristic distribution pattern, with the IR regions being highly conserved, whereas the LSC and SSC regions showed relatively higher levels of sequence variation. Most regions exhibited Pi values below 0.01, indicating that the cp genomes of the 16 samples were generally conserved, with significant sequence divergence restricted to a limited number of loci. A total of 12 highly variable regions were identified, most of which were located within the LSC and SSC regions, whereas highly divergent sites were rarely detected in the IR regions. Among these hotspots, the *trnH-GUG-psbA* IGS exhibited the highest nucleotide diversity (Pi = 0.02415), representing the most divergent region among the analyzed samples. The *ycf1* gene, *petN-psbM* IGS, and *trnD-GUC-trnY-GUA* IGS showed the next highest levels of nucleotide diversity, with Pi values of 0.01524, 0.01425, and 0.01417, respectively. In addition, the IGS of *trnK-UUU-rps16、rpl32-ccsA、psbE-petL、petA-psbJ、psaJ-rpl33、trnF-GAA-ndhJ*, as well as the *rps8* gene, also exhibited relatively high nucleotide diversity among the 16 samples, with Pi values exceeding 0.01 (**Figure 9**).

**Figure 9:**
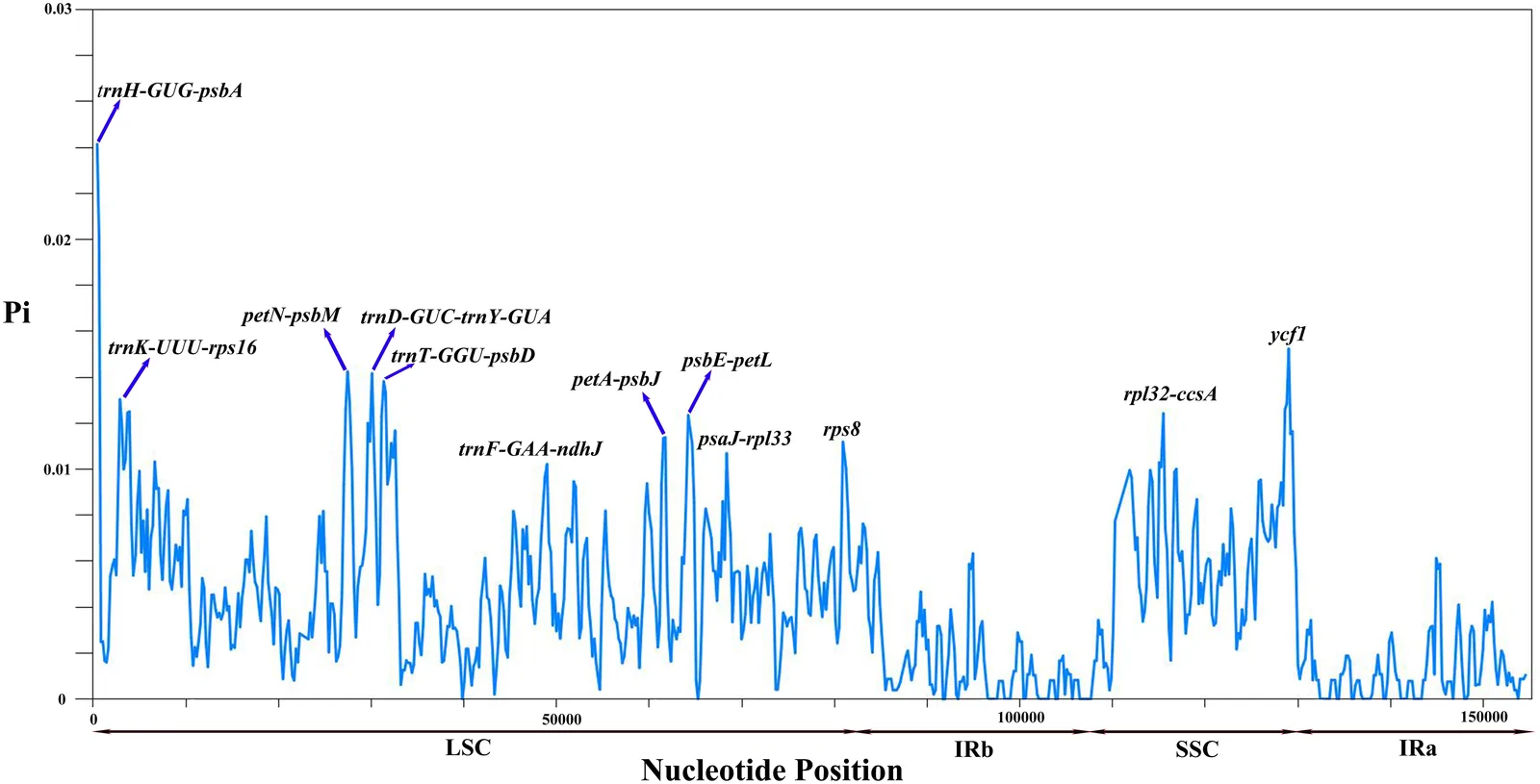
Highly variable regions identified in the chloroplast genomes of 16 samples.

### 3.6 Phylogenetic analysis

Phylogenetic analyses were conducted using the complete cp genome sequences of 16 samples from Sect. *Petiolares* and 31 closely related taxa within *Primula*, with *Lysimachia chapaensis* (Primulaceae, *Lysimachia*) and *Androsace laxa* (Primulaceae, *Androsace*) selected as outgroups. Phylogenetic relationships were inferred using both the ML and BI methods. The phylogenetic trees generated by the two approaches were largely congruent in terms of the major clades; however, incongruences were observed in the phylogenetic placement of *P. taliensis* and the relative positions of the *P. sonchifolia* and *P. strumosa* lineages within the section (**Figures 10**, **11**). In the ML tree, most internal nodes received strong bootstrap support, with the majority of nodes showing bootstrap values (BS) of 100%. Nevertheless, several nodes exhibited comparatively lower support values, including the node corresponding to the *P. ovalifolia* complex clade (BS = 76), some intraspecific relationships within *P. esquirolii* (BS = 91-100), and the node representing the paraphyletic relationship between (*P. hookeri*, *P. panzhouensis*) and (*P. sonchifolia*, *P. strumosa*) (BS = 94). In contrast, the BI tree showed maximum posterior probability support (PP = 1.0) for all nodes, indicating consistently high confidence in the inferred phylogenetic relationships.

**Figure 10:**
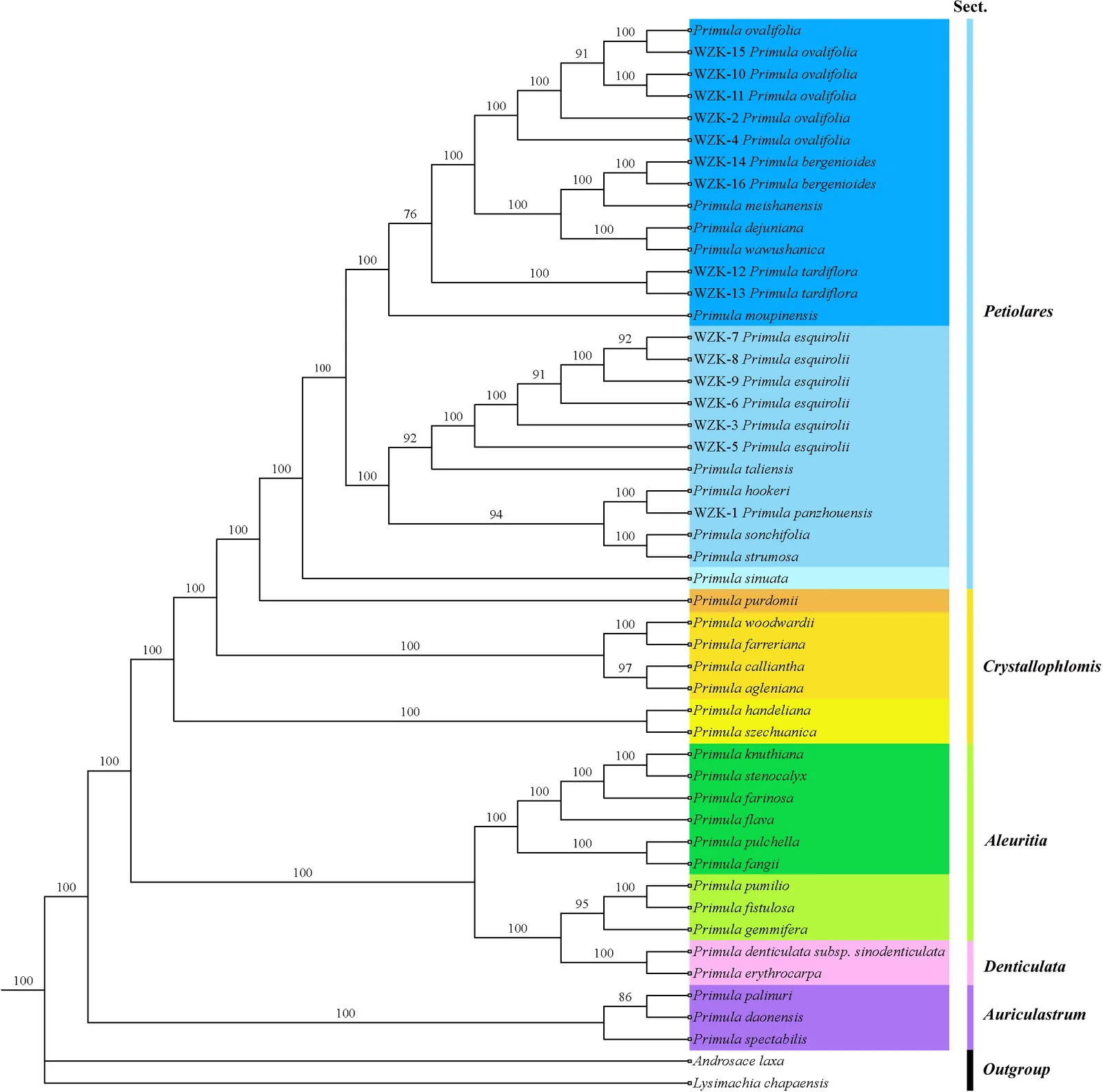
Phylogenetic tree constructed using maximum likelihood (ML) method based on complete chloroplast genome sequences.

**Figure 11:**
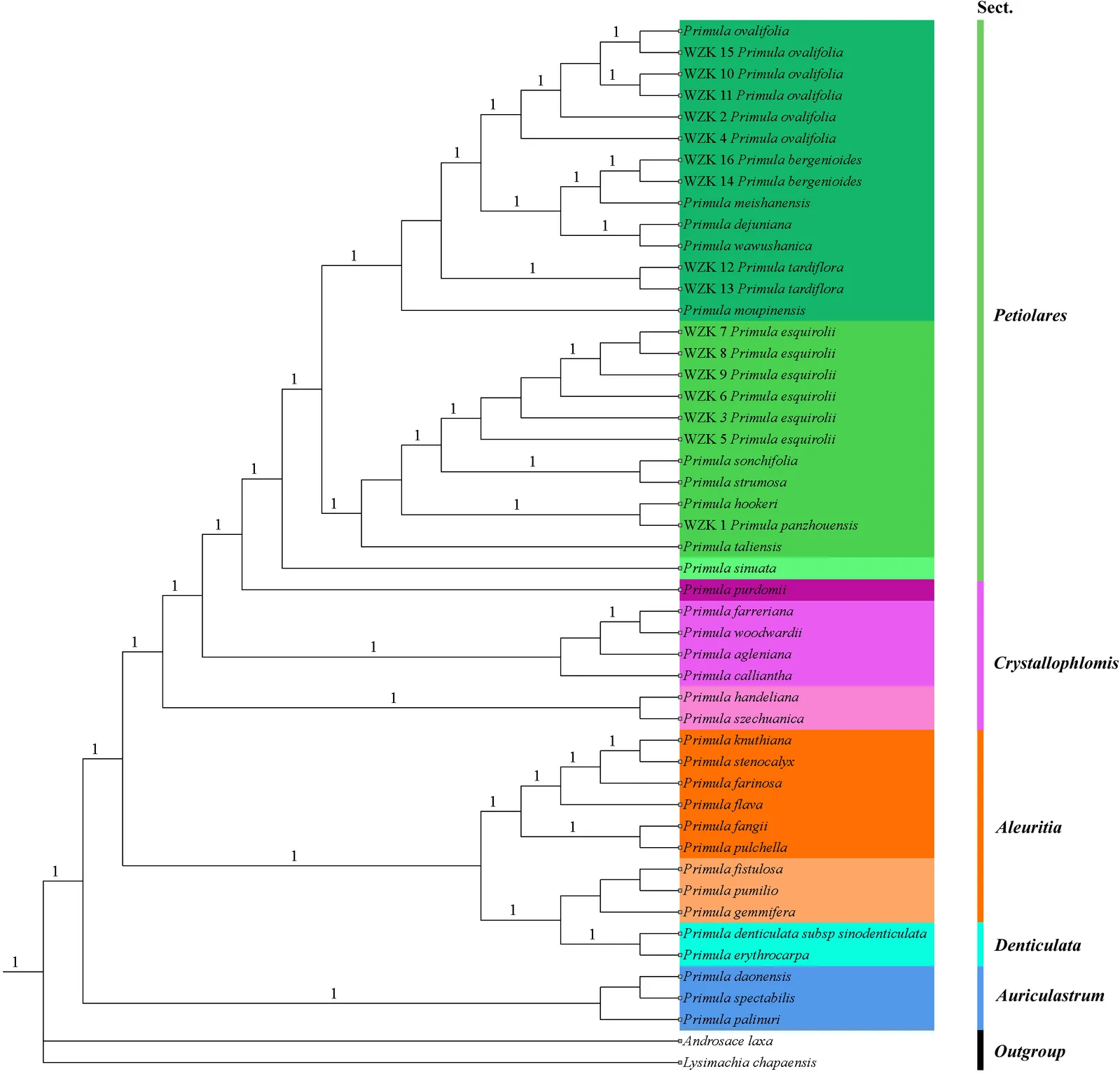
Phylogenetic tree constructed using bayesian inference (BI) method based on complete chloroplast genome sequences.

Clustering analyses indicated that all *Primula* species formed a monophyletic clade, with the divergence node separating the ingroup from the outgroup receiving 100% bootstrap support and a posterior probability of 1.0. Within the genus, species were grouped into five major clades corresponding to traditionally recognized taxonomic sections, each of which formed an independent monophyletic lineage. Among these, Sect. *Auriculasrum* constituted a well-supported monophyletic group and occupied a basal position within the main *Primula* clade, representing one of the earliest diverging lineages in the genus. The topology of this section was clear and stable, and it formed a sister relationship with the remaining four sections.

Within Sect. *Petiolares*, both the ML and BI analyses consistently recovered *P. sinuata* as the earliest-diverging lineage. *P. sinuata* was positioned at the basal position of the section and formed a strongly supported sister relationship with all remaining taxa, indicating that it represents the most basal lineage within Sect. *Petiolares*. The remaining taxa were further resolved into two major clades. The first clade comprised the putative new species WZK-1 *P. panzhouensis*, *P. hookeri*, *P. sonchifolia*, *P. strumosa*, *P. taliensis*, and the *P. esquirolii* complex. Within this clade, the putative new species WZK-1 *P. panzhouensis* formed a direct sister relationship with *P. hookeri*. *P. sonchifolia* and *P. strumosa* were recovered as immediate sister taxa, and together they formed a strongly supported monophyletic lineage. This lineage was subsequently resolved as sister to the *P. esquirolii* complex. The phylogenetic position of *P. taliensis* showed slight incongruence between the two phylogenetic frameworks. In the ML tree, *P. taliensis* was recovered as sister to the *P. esquirolii* complex, whereas in the BI tree, it was resolved as sister to the aforementioned taxa and occupied a basal position within the first major lineage. The second clade consisted of several sampled taxa belonging to Sect. *Petiolares* subsect. *Davidii*, including *P. ovalifolia*, *P. bergenioides*, *P. meishanensis*, *P. dejuniana*, *P. wawushanica*, *P. tardiflora*, and *P. moupinensis*. Multiple geographically distinct samples of *P. ovalifolia* were recovered as a monophyletic lineage. The relationships among the lineages within Sect. *Petiolares* were clearly resolved, and the recovered phylogenetic topology was highly stable and well supported. These results demonstrate that Sect. *Petiolares* represents a strongly supported monophyletic group. Furthermore, the close phylogenetic affinity between the putative new species from the Panzhou population and other samples within Sect. *Petiolares* indicates that this taxon should be assigned to Sect*. Petiolares*.

The sister relationship between the putative new species *P. panzhouensis* and *P. hookeri* was strongly supported in both phylogenetic analyses (BS = 100, PP = 1.0). Based on the inferred phylogenetic relationships and comparative analyses of cp genomes, the WZK-1 *P. panzhouensis* sample represents a distinct lineage from all other known samples of Sect. *Petiolares* within the currently available sampling framework. Together with the observed morphological differences and its phylogenetic distinctiveness, these results provide strong evidence supporting its recognition as a new species. According to the geographical distribution of the type specimen, the putative new species is herein named *Primula panzhouensis* Z.K. Wu, sp. nov.

### 3.7 Morphological description

#### 3.7.1 Taxonomic treatment

*Primula panzhouensis* Z.K.Wu, sp. nov. (**Figures 12**, **13**).

**Figure 12:**
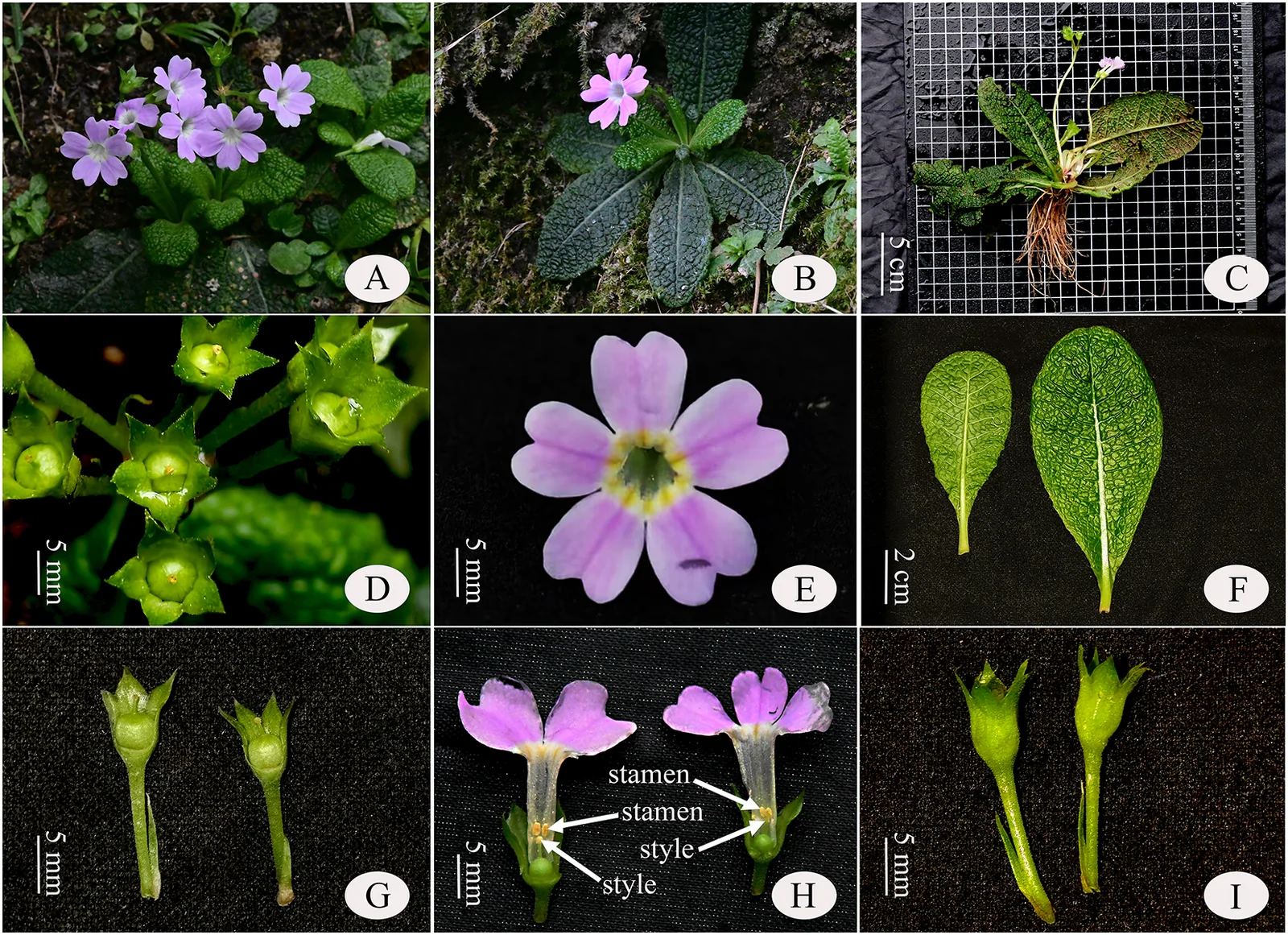
**(A–I)** *Primula panzhouensis* sp. nov. **(A)** habitat; **(B)** habit in late flowering; **(C)** specimen in flowering; **(D)** capsule in early-fruiting; **(E)** flower, front view; **(F)** leaves, right: upper surface, left: lower surface; **(G)** calyx and stigma; **(H)** dissected corolla showing the anthers and stigma; **(I)** calyx and bract. Photographed by Z.K.Wu.

**Figure 13:**
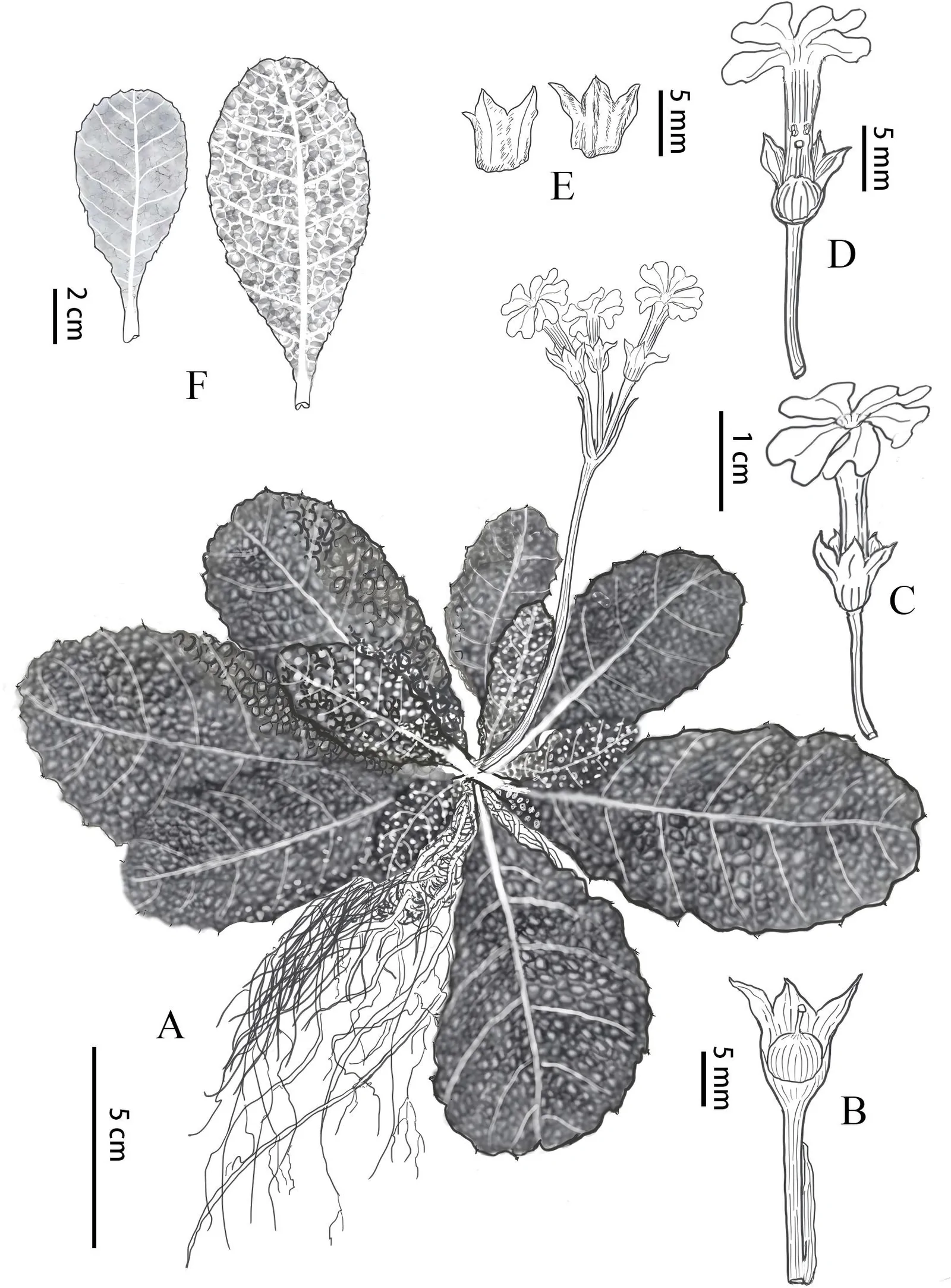
*Primula panzhouensi*s sp. nov. **(A)** habit; **(B)** capsule; **(C)** calyx and corolla; **(D)** dissected corolla showing the anthers and stigma; **(E)** calyx; **(F)** leaves, right: upper surface, left: lower surface. Drawn by Ms. Xiang-Li Wu.

#### 3.7.2 Type

China. Guizhou: Panzhou county, Jichangping, 25.987501°N, 104.619186°E, 1754 m, 16 March, 2025, ZKWU20250316001 (holotype: KUN)!.

#### 3.7.3 Diagnosis

The new species is morphologically similar to *P. esquirolii*, *P. bergenioides*, and *P. hookeri*, and all four species exhibit morphological characteristics consistent with the diagnostic features of Sect. *Petiolares*. It differs from *P. esquirolii* primarily in that the latter produces few or no new leaves during the flowering period, has a relatively long corolla tube, and bears extremely shortened scapes that are usually less than 1 cm in height or nearly absent. In addition, *P. esquirolii* is homostylous with long-style flowers, whereas the new species possesses a short corolla tube, well-developed scapes, and produces numerous new leaves during anthesis. Compared with the heterostylous *P. bergenioides*, the new species is readily distinguished by its short-styled homostylous flowers, whereas *P. bergenioides* exhibits a typical heterostylous floral system. The new species can be distinguished from *P. hookeri* mainly by several morphological and ecological characters. *P. hookeri* occurs at higher elevations, generally above 3,800 m, and lacks overwintering leaves, with all mature leaves withering during winter. Furthermore, its scapes are absent during the flowering period, and the abaxial surface of the leaves lacks honeycomb-like protuberances. In contrast, the new species grows at a much lower elevation (approximately 1,700-1,800 m), retains overwintering mature leaves, develops elongated scapes during anthesis, and possesses distinct honeycomb-like protuberances on the abaxial leaf surface (**Figures 12**, **14**). **Table 5** summarizes the main morphological differences among *P. panzhouensis*, *P. esquirolii*, *P. bergenioides*, and *P. hookeri*.

**Figure 14:**
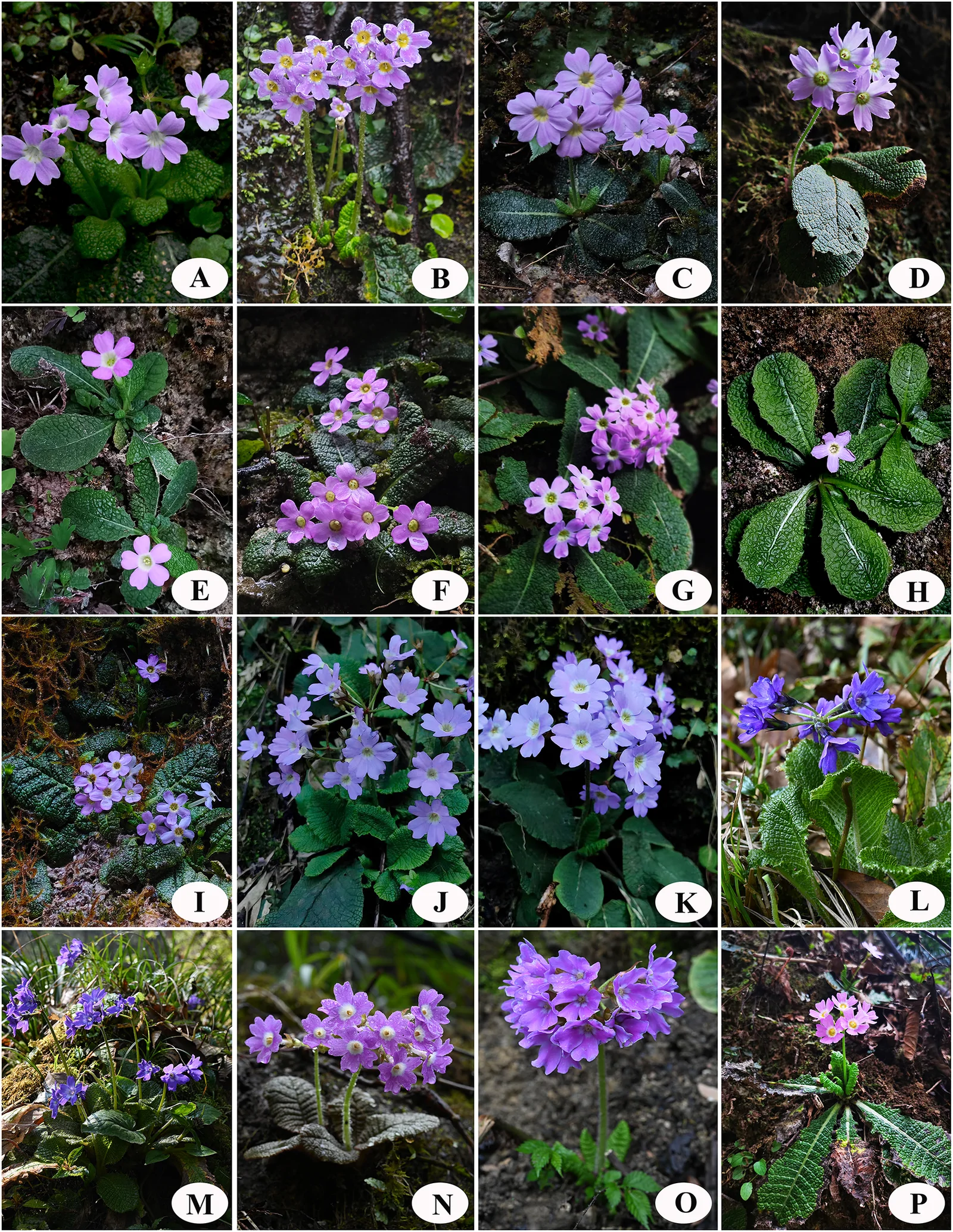
Plant photographs of 16 samples. **(A)** WZK-1 *P. panzhouensis;* **(B)** WZK-2 *P. ovalifolia;* **(C)** WZK-3 *P. esquirolii*; **(D)** WZK-4 *P. ovalifolia*; **(E)** WZK-5 *P. esquiroli*i; **(F)** WZK-6 *P. esquirolii*; **(G)** WZK-7 *P. esquirolii*; **(H)** WZK-8 *P. esquirolii;* **(I)** WZK-9 *P. esquiroli*i; **(J)** WZK-10 *P. ovalifolia*; **(K)** WZK-11 *P. ovalifolia*; **(L)** WZK-12 *P. tardiflora*; **(M)** WZK-13 *P. tardiflora*; **(N)** WZK-14 *P. bergenioides*; **(O)** WZK-15 *P. ovalifolia*; **(P)** WZK-16 *P. bergenioides*.

**Table 5:** Morphological comparisons of *P. panzhouensis* with *P. esquirolii, P. bergenioides* and *P. hookeri*.

Characters	*P. panzhouensis*	*P. esquirolii*	*P. bergenioides*	*P. hookeri*
Scape	Flowering period is noticeable, height 5.5-10.5 cm	Almost obsolete or to 1 cm in flowering time	Height 13–20 cm, 1 to 2 per plant, gla-brous, carrying a terminal umbel of 5 to 11 flowers	Less than 3 mm, included in leaves, elongating to 30 cm in fruit
Leaf blade	5.2-9.5 × 2.1-3.5cm, elliptic-obovate to obovate-oblanceolate, apex rounded, base attenuate, leathery, glabrous, margin shallowly undulate-serrate, adaxially bullate, abaxially honeycomb-like	5-13 × 1.5–5 cm, elliptic-obovate to obovate-oblanceolate, subcoriaceous, abaxially densely short glandular pubescent along veins, adaxially bullate	12-20 × 6–9 cm, leaf blade oblong-elliptic to elliptic-obovate, bullate on the margin and covered with minute glandular hairs, young leaves bullate and glandular-hairy, soon glabrescent, and finally smooth and glabrous on both surfaces	1.5-4 × 0.6-1.5 cm at anthesis, to 15 × 5 cm at fruiting, subsessile and slightly emergent from basal scales at anthesis, distinctly petiolate in fruit, leaf blade oblong-obovate to spatulate, tapering to base, apex rounded to obtuse
Calyx	4-7mm long	5–7 mm long	10-12mm long	5–8 mm long
Calyx lobes	Parted to about 1/3 of full length	Parted to about 2/5 of full length	Parted to about 1/3 of full length	Parted nearly to middle
Flower	Short-homostylous; stigmas and anthers situated at the base of the corolla tube. Corolla limb 6–13 mm in diameter; corolla tube 7–12 mm long.	Long-homostylous; stigmas and anthers positioned at the mouth of the corolla tube. Corolla limb 15–20 mm in diameter; corolla tube 15–16 mm long.	Heterostylous; both pin and thrum morphs present, with reciprocal positioning of the stigma and stamens. Corolla limb 18–25 mm in diameter; corolla tube ca. 13 mm long.	Homostylous; stamens inserted at middle of corolla tube, style slightly longer than anthers. Corolla limb 7–10 mm in diameter; corolla tube 7–8 mm long.

#### 3.7.4 Description

Perennial herbs, with a stout and short rhizome and numerous fibrous adventitious roots. At anthesis, a ring of reddish-brown scales is present at the basal periphery of the leaf rosette. Leaves elliptic-obovate to oblanceolate, glabrous; mature leaves dark green, younger leaves light green; leaf blades 5.2-9.5 cm long and 2.1-3.5 cm wide; apex rounded, base gradually attenuate; margin shallowly undulate-serrate. When dry, the blades become subcoriaceous; adaxial surface bullate due to sunken reticulate veins, sparsely covered with short-stalked glandular trichomes; abaxial surface with prominent lateral veins and reticulate venation, giving a reticulate or honeycomb-like appearance, veins also bearing short-stalked glandular trichomes. Petioles usually indistinct or absent. Scapes 5.5-10.5 cm tall at flowering, sparsely covered with short-stalked glandular trichomes in the upper portion. Umbels 3-7-flowered. Bracts narrowly lanceolate, gradually tapering from a slightly broadened base, 4–7 mm long, submembranous. Pedicels 6–11 mm long, glandular-pubescent with short-stalked trichomes. Calyx campanulate, basally inflated, approximately two-thirds the length of the pedicel, 4–7 mm long; outer surface sparsely covered with minute glands; calyx divided to about one-third of its length; lobes triangular-lanceolate, apex slightly attenuate. Corolla pale purple to purple; limb 6–13 mm in diameter; corolla tube tubular, 7–12 mm long, throat lacking annular appendages; lobes 2–4 mm in diameter, obovate, apically deeply emarginate. Flowers short-styled homostylous; stamens inserted at the base of the corolla tube, nearly equaling the style in height. Capsule globose, enclosed within the calyx tube.

#### 3.7.5 Distribution and habitat

This new species is currently only known from the type locality on Jichangping in Panzhou county, Guizhou, China, and is mostly found on damp cliffs of karst formations, at altitudes of 1700–1800 m (**Figure 15**).

**Figure 15:**
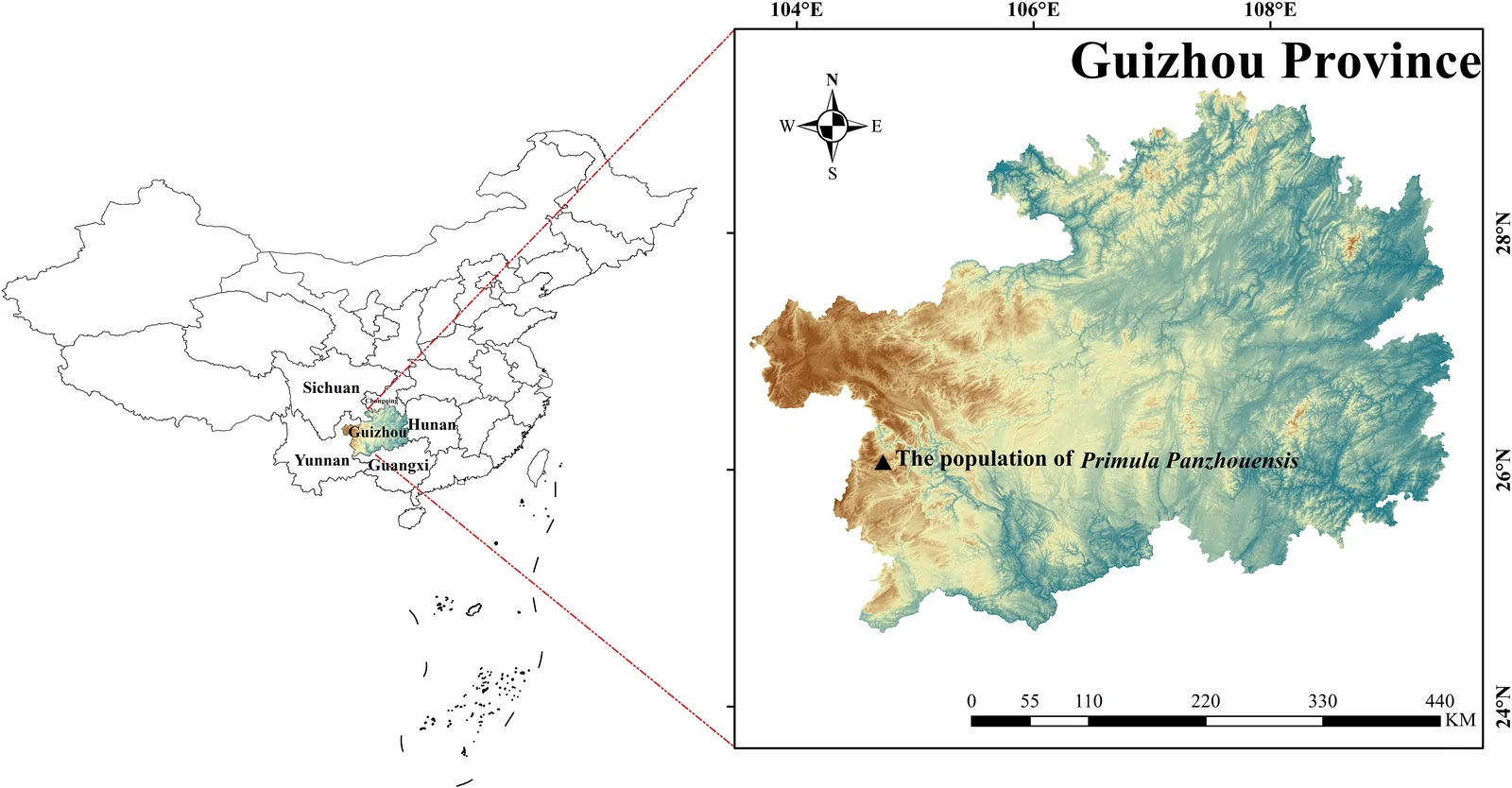
Location of the distribution of *P. panzhouensis* in Panzhou, Guizhou.

#### 3.7.6 Phenology

The species was observed to flower from January to March, fruiting from April to June.

#### 3.7.7 Etymology

The specific epithet of the new species is taken from the Chinese Pinyin “Panzhou”, the name of the county in western Guizhou, China, where the type specimen was collected (**Figure 15**).

#### 3.7.8 Vernacular name

Chinese mandarin: pán zhōu bào chūn (盘州报春).

#### 3.7.9 Provisional conservation status

This species exhibits a highly restricted distribution range. Field surveys were conducted several times in the type locality and adjacent districts for this new species, and only one population of *P. panzhouensis* was discovered, with ca. 100 adult individuals, distributed over about 100 m^2^ in the type locality. This site is on the steep cliff near a temple, where its limited seed dispersal range severely restricts its capacity to adapt to environmental changes, and some individuals grow close to the path for visitors and face a strong threat from human activities. Consequently, its conservation status warrants urgent attention, and targeted protection measures should be developed through further investigations. We estimated the extent of occurrence of the species to be less than 100 km^2^. Over the past four years, we have documented a continuous decline in its habitat range driven by multiple compounding pressures. Temple maintenance and road construction projects have progressively encroached upon the remaining habitat, exacerbating the species’ vulnerability. Therefore, in combination with the information currently available and in accordance with the guidelines for the use of the IUCN Red List Categories and Criteria, the conservation status of this new species has been assessed as ‘Critically Endangered’ (CR B2ab(iii)).

## 4 Discussion

### 4.1 Effectiveness of chloroplast comparative genomics and phylogenetic analyses in cryptic species delimitation

Comparative analysis of complete cp genomes from 16 samples of Sect. *Petiolares*, combined with phylogenetic relationships and morphological evidence, provided multiple lines of evidence supporting the taxonomic treatment of *P. panzhouensis*. Comparative cp genomic analyses revealed that *P. panzhouensis* differed from its closely related samples in several genomic features, including IR boundary configurations, SSR composition, and sequence variations in several highly variable regions. These results suggest that this lineage possesses recognizable cp genomic characteristics. However, it should be emphasized that only a single cp genome of *P. panzhouensis* was obtained in the present study. Therefore, these differences should be interpreted as characteristics of the currently sequenced individual rather than fixed genomic features shared by all populations of this species. Additional sampling of multiple individuals will be necessary to evaluate the stability of these structural variations at the intraspecific level. Phylogenetic analyses based on complete cp genomes using both ML and BI methods consistently placed *P. panzhouensis* within Sect. *Petiolares*, forming an independent chloroplast monophyletic lineage with strong support and exhibiting a stable sister relationship with *P. hookeri*. Combined with its distinctive morphological characteristics, including short-styled homostyly, elongated scapes, and unique leaf traits, these results support the recognition of this lineage as an independent taxonomic entity.

Similarly, increasing evidence from recent studies indicates that cp genomes can provide valuable lineage-resolving information and facilitate the identification of morphologically cryptic taxa in rapidly radiating groups (Song et al., 2022). However, cp genomes represent only a single genetic system, and their evolutionary histories may be influenced by processes such as chloroplast capture, historical introgression, and incomplete lineage sorting. Therefore, the phylogenetic independence of *P. panzhouensis* and the evolutionary history of its divergence from closely related taxa require further investigation using nuclear genomic data and population-level analyses.

### 4.2 Evolutionary patterns and ecological implications of short-homostyly in *Primula*

*Primula* has long been regarded as a classical model system for investigating the evolution of heterostyly. The *S*-locus supergene and its associated regulatory components, particularly *CYP734A50*, play central roles in maintaining floral dimorphism (Huu et al., 2016, Huu et al., 2022). Previous studies have demonstrated that degeneration or loss of *S*-locus function can repeatedly give rise to homostyly, accompanied by complex shifts in reproductive strategies and adaptive evolution (Li et al., 2016). Nevertheless, short-styled homostylous forms have generally been considered unlikely to persist in natural populations because the reduced spatial correspondence between stigmas and anthers is expected to limit pollination efficiency. Consequently, naturally occurring populations of short-styled homostylous taxa have often been interpreted as transient degenerative forms or the result of anthropogenic disturbance, as exemplified by *P. vulgaris* (Li et al., 2016). Consistent with this view, our long-term field investigations indicate that long-styled homostylous species are overwhelmingly predominant among homostylous *Primula*, whereas short-styled homostylous species are exceptionally rare. To date, we have observed only one additional unpublished species in Sect. *Aleuritia* that maintains stable natural populations of the short-styled homostylous form (Z.K. Wu, unpublished data).

The present study revealed that *P. panzhouensis* possesses a stable short-styled homostylous floral phenotype. During nearly four consecutive years of field observations, no distylous individuals were detected, and all examined individuals consistently exhibited the short-styled homostylous condition. In addition, observations of seeds collected under natural conditions demonstrated that this lineage is capable of producing seeds in its native habitat. These findings indicate that short-styled homostyly in *P. panzhouensis* is not merely an occasional individual variation, but rather a floral condition that can persist in natural populations capable of completing reproduction. Previous studies have suggested that the origin of homostyly is generally associated with alterations in the mechanisms maintaining heterostyly (Li et al., 2016). Research on other *Primula* lineages has shown that the emergence of short-styled homostyly may be related to modifications of functional modules within the *S*-locus. For example, studies of *CYP734A50* have demonstrated that loss of this gene can disrupt the maintenance of reciprocal style-length dimorphism, thereby promoting transitions toward homostyly (Huu et al., 2016, Huu et al., 2022). Furthermore, the *S*-locus supergene exhibits complex patterns of structural rearrangement and expression divergence among different lineages (Li et al., 2016), suggesting that the evolution of floral polymorphism is highly flexible. However, whether the short-styled homostyly of *P. panzhouensis* is associated with structural changes in the *S*-locus remains to be determined through further genomic and functional investigations. Recent studies on ecological differentiation within Chinese *Primula* have indicated that different floral morphs, including heterostylous and homostylous forms, exhibit distinct patterns of spatial distribution and ecological adaptation, with homostylous taxa frequently occurring in relatively extreme or isolated microhabitats (Li et al., 2024). This pattern is consistent with the habitat characteristics of *P. panzhouensis*, which occurs on karst cliff environments characterized by strong habitat isolation and specialized ecological conditions.

Classical studies on the origin of heterostyly have proposed that the evolution of this reproductive system represents an adaptation for enhancing pollination accuracy, known as the “pollination-precision hypothesis,” and that similar floral strategies have evolved repeatedly across angiosperms through convergent evolution (Barrett, 1990). Therefore, the occurrence of short-styled homostyly in *P. panzhouensis* may represent an alternative floral strategy during the diversification of reproductive systems in *Primula*, rather than simply reflecting a degenerative evolutionary process.

*P. panzhouensis* provides an important system for further investigating the origin of short-styled homostyly, its genetic basis, and its potential relationship with structural evolution of the *S*-locus. It should be emphasized that the present study demonstrates the persistence of short-styled homostyly under natural conditions and the ability of this floral type to achieve seed production, but does not establish that this phenotype has evolved a long-term adaptive advantage. Future studies integrating *S*-locus structural analyses, comparisons of selfing and outcrossing fitness, pollen competition experiments, and population genetic investigations will be essential for elucidating the evolutionary mechanisms underlying this specialized reproductive strategy.

### 4.3 Karst habitat heterogeneity and its role in speciation

The karst regions of southwestern China are characterized by highly complex topographic structures and diverse microhabitats, making them one of the global hotspots for the formation of plant endemism (Oliver et al., 2017; Xie et al., 2021). Previous studies have demonstrated that spatial isolation and environmental heterogeneity can promote population divergence and influence local adaptation processes (Xie et al., 2021; Liang et al., 2022). Within the broader evolutionary framework of *Primula*, the complex topographic patterns and climatic gradients of southwestern China have been recognized as important drivers of diversification within this genus (Xie et al., 2012; Huang et al., 2026). In the present study, the newly described species *P. panzhouensis* is currently known only from a restricted karst humid cliff habitat in Panzhou, Guizhou Province. Its extremely narrow distribution range, single known population, and specialized microhabitat are consistent with the highly heterogeneous environmental conditions characteristic of karst ecosystems. However, the current evidence remains insufficient to directly demonstrate that karst environments have driven the origin and diversification of *P. panzhouensis*. Comprehensive investigations involving population genetic structure analyses, gene flow assessments, historical demographic reconstructions, and ecological niche differentiation experiments are still lacking. Therefore, we propose that the localized distribution pattern of *P. panzhouensis* suggests a potential role of karst environments in shaping its lineage divergence, although this hypothesis requires further validation through future population genetic and ecological studies.

Furthermore, this study revealed that only short-styled homostylous individuals occur in natural populations of *P. panzhouensis*, a condition that differs from the typical distylous system commonly observed in *Primula*. However, because direct experiments on pollination biology, self-compatibility, mating systems, and reproductive ecology were not conducted in the present study, it remains unclear whether this floral condition represents a stable reproductive system or merely reflects a phenotypic response under localized environmental conditions. Accordingly, we treat short-styled homostyly as an important morphological and reproductive characteristic observed in this taxon, while the evolutionary stability of its reproductive system requires further evaluation through controlled crossing experiments, seed germination studies, and analyses of floral morph segregation patterns in subsequent generations.

### 4.4 Limitations and future perspectives

This study integrated cp genomic and morphological evidence to support the recognition of *P. panzhouensis* as an independent taxonomic entity and documented its distinctive short-styled homostylous floral trait. However, several limitations remain. At present, only a single cp genome of *P. panzhouensis* has been obtained, and whether some of the observed genomic structural features represent stable intraspecific characteristics requires validation through additional sampling of multiple individuals. Moreover, cp genomes represent only a maternally inherited genetic component and may not fully reflect the evolutionary history of the nuclear genome.

Future studies incorporating population-level nuclear genomic data, such as RAD-seq and ddRAD approaches, together with nuclear markers including ITS and single-copy nuclear genes, will provide further insights into the demographic history, gene flow patterns, and divergence processes between *P. panzhouensis* and its closely related taxa. In addition, investigations of *S*-locus architecture and reproductive ecological traits will be essential for elucidating the genetic mechanisms underlying the origin and maintenance of short-styled homostyly.

## 5 Conclusions

In this study, we conducted an integrative investigation of a newly discovered taxon of *Primula* from the karst region of Panzhou, Guizhou Province, southwestern China, based on comparative analyses of complete cp genomes, phylogenetic reconstruction, and morphological evidence. The cp genome of this new species exhibited a typical quadripartite structure and was highly conserved with other taxa of Sect. *Petiolares* in terms of overall genome organization and gene composition. However, several differences were detected in specific IR boundary regions, repeat sequence composition, and highly variable regions. Phylogenetic analyses based on complete cp genomes using both ML and BI approaches consistently supported the placement of *P. panzhouensis* within Sect. *Petiolares* and revealed that it represents an independent chloroplast lineage. Combined with its unique and stable morphological characteristics, including short-styled homostyly, elongated scapes, relatively short corolla tubes, and the presence of newly emerging leaves during the flowering period, our results support the recognition of this lineage as an independent taxonomic entity, here described as *Primula panzhouensis* Z.K.Wu, sp. nov.

Notably, *P. panzhouensis* represents an extremely rare short-styled homostylous form within *Primula*. Nearly four years of continuous field observations revealed that all individuals within the known natural population exhibited short-styled homostyly, with no distylous individuals observed. Furthermore, observations of naturally produced seeds indicated that this species is capable of completing seed formation under natural conditions. These findings demonstrate that short-styled homostyly can persist in natural populations and support successful reproduction, providing valuable material for understanding the diversity of floral polymorphisms and the evolution of reproductive strategies in *Primula*. However, the evidence presented in this study is primarily derived from cp genomes, morphological comparisons, and field observations of reproduction, and therefore does not fully resolve the evolutionary history of the nuclear genome, population genetic structure, or the genetic mechanisms underlying the origin and maintenance of short-styled homostyly. Future studies integrating nuclear genomic data and reproductive ecological analyses will be essential for further elucidating the evolutionary processes shaping this specialized reproductive strategy.

At present, *P. panzhouensis* is known only from a restricted humid karst cliff habitat in Panzhou, Guizhou Province. This narrow distribution pattern suggests that heterogeneous karst environments may have contributed to its localized divergence; however, the specific evolutionary role of karst habitats requires further investigation.

This study not only expands species diversity and cp genomic resources within *Primula*, but also provides a valuable foundation for investigating rare reproductive strategies, taxonomic delimitation, and the processes underlying plant diversification in the karst regions of southwestern China.

## Data Availability

The datasets presented in this study can be found in online repositories. The names of the repository/repositories and accession number(s) can be found in the article/**Supplementary Material**.

## Supplementary material

Supplementary Figure 1 Chloroplast genome maps of 15 Sect. *Petiolares* samples.
